# Supplementary material for: Mitochondrial NAD+ Controls Nuclear ARTD1-Induced ADP-Ribosylation
Source: Mol Cell. 2021 Jan 21;81(2):340–354.e5. doi: 10.1016/j.molcel.2020.12.034 (PMC7837215; doi:10.1016/j.molcel.2020.12.034)
Supplement: Document S1. Figures S1–S7 [file mmc1.pdf]

Molecular Cell, Volume *81*

Supplemental Information

Mitochondrial NAD^+ Controls Nuclear ARTD1-Induced ADP-Ribosylation

Ann-Katrin Hopp, Federico Teloni, Lavinia Bisceglie, Corentin Gondrand, Fabio Raith, Kathrin Nowak, Lukas Muskalla, Anna Howald, Patrick G.A. Pedrioli, Kai Johnsson, Matthias Altmeyer, Deena M. Leslie Pedrioli, and Michael O. Hottiger

Suppl. Figure 1

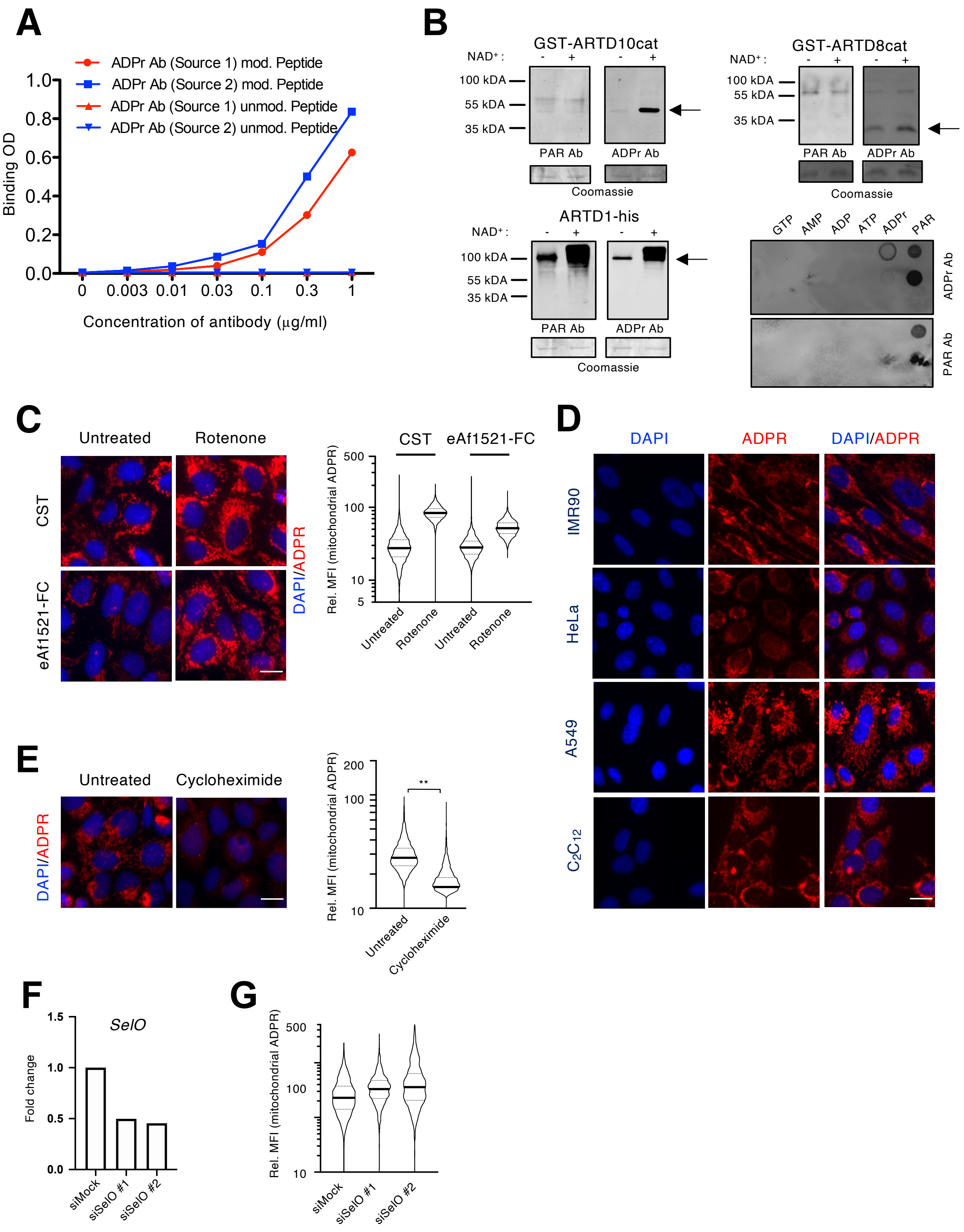

Suppl. Figure 1: Validation of the new pan-anti-ADP-ribosylation antibody, related to Figure 1

(**A**) ELISA using the two anti ADPr antibodies (source 1 and source 2) in titrating concentrations on two different modified and unmodified peptides. (**B**) WB of the auto-modified catalytic domain of ARTD10 (ARTD10-cat), or ARTD8 (ARTD8-cat) or auto-modified ARTD1 stained with the anti-PAR antibody and the anti-ADPr antibody. Dot blot with two concentrations (10 and 1 μM; upper and lower row, respectively) of GTP, AMP, ADP, ATP, ADPr and PAR, stained with the anti-ADPr antibody or an anti-PAR antibody (lower right panel). (**C**) IF staining of U2OS cells either untreated or treated with 3 μM Rotenone using either a commercial anti-pan-ADPr antibody or an engineered Af1521-FC fusion protein. (**D**) IF staining using the anti-ADPr antibody (red) on C2C12 myoblasts, HeLa, A549 and IMR90. (**E**) IF staining for ADP-ribosylation in cells pre-treated with cycloheximide. (**F**) qPCR analysis of the knockdown levels of SelO. (**G**) Quantification of mitochondrial ADP-ribosylation in U2OS after knockdown of SelO with two independent siRNAs. For the representation of all quantifications the signal for every event was normalized over the mean of the control/untreated sample, which was arbitrarily set to 30. The Y-axes of all violin plots are depicted as log 10 scales. Scale bars indicate 20 μm. For statistical analysis, a Student's t test was performed ($n = 3\text{-}5$; $*p < 0.05$; $**p < 0.005$; $***p < 0.0005$).

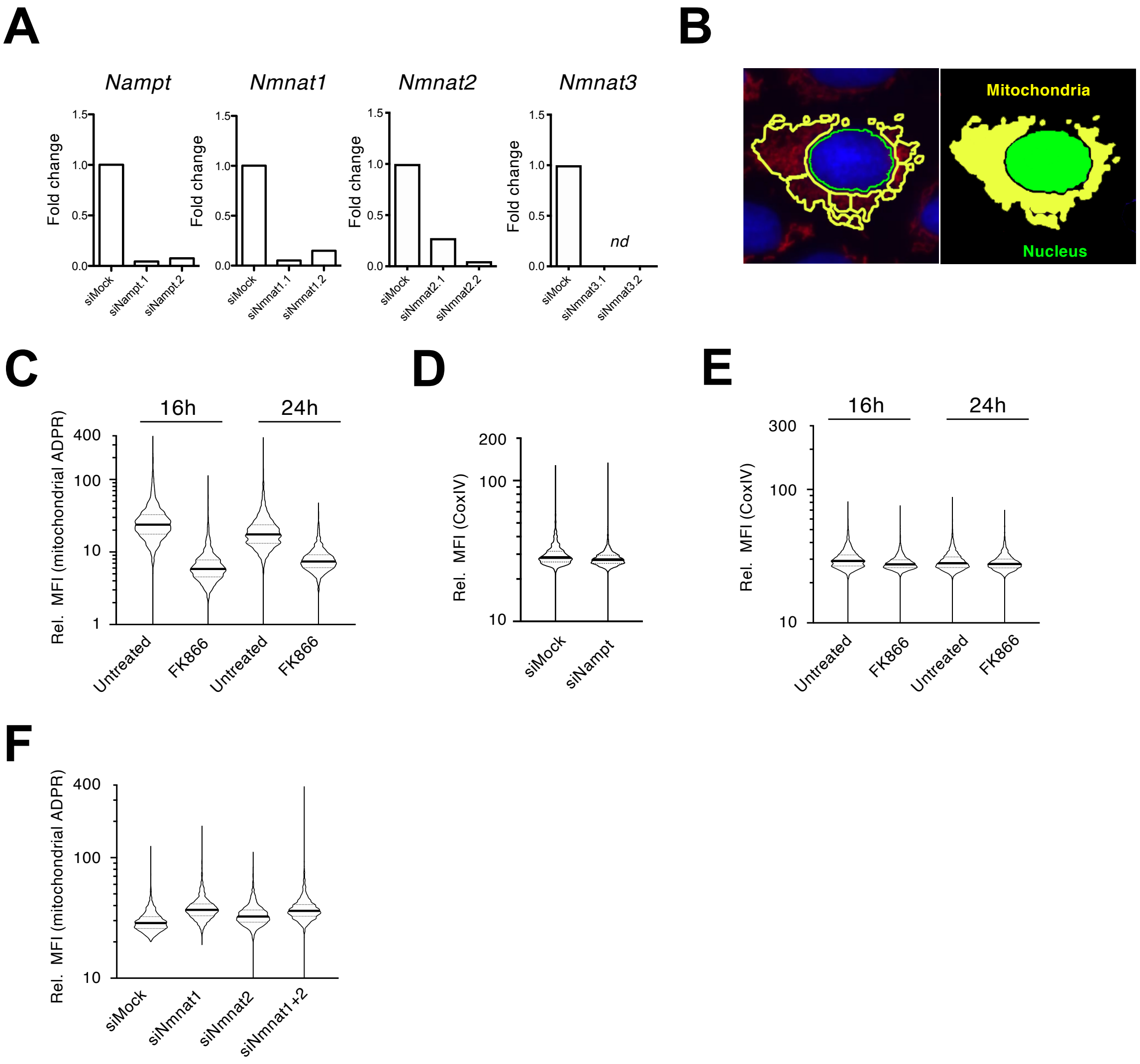

Suppl. Figure 2: Characterization of mitochondrial ADP-ribosylation, related to Figure 1

(A) qPCR analysis of the knockdown levels of NAMPT, NMNAT1, NMNAT2 and NMNAT3 after siRNA mediated knockdown of the respective genes in U2OS cells. **(B)** Scheme of the masks applied to quantify nuclear- as well as extra-nuclear IF signals. **(C)** Quantification of mitochondrial ADP-ribosylation after treatment with the NAMPT inhibitor FK866 for 16 or 24h. **(D,E)** Quantification of the mitochondrial COXIV signal in U2OS cells after knockdown of NAMPT or treatment with FK866. **(F)** Quantification of mitochondrial ADP-ribosylation in U2OS cells after knockdown of NMNAT1, NMNAT2 or both. For the representation of all quantifications the signal for every event was normalized over the mean of the control/untreated sample, which was arbitrarily set to 30. The Y-axes of all violin plots are depicted as log 10 scales.

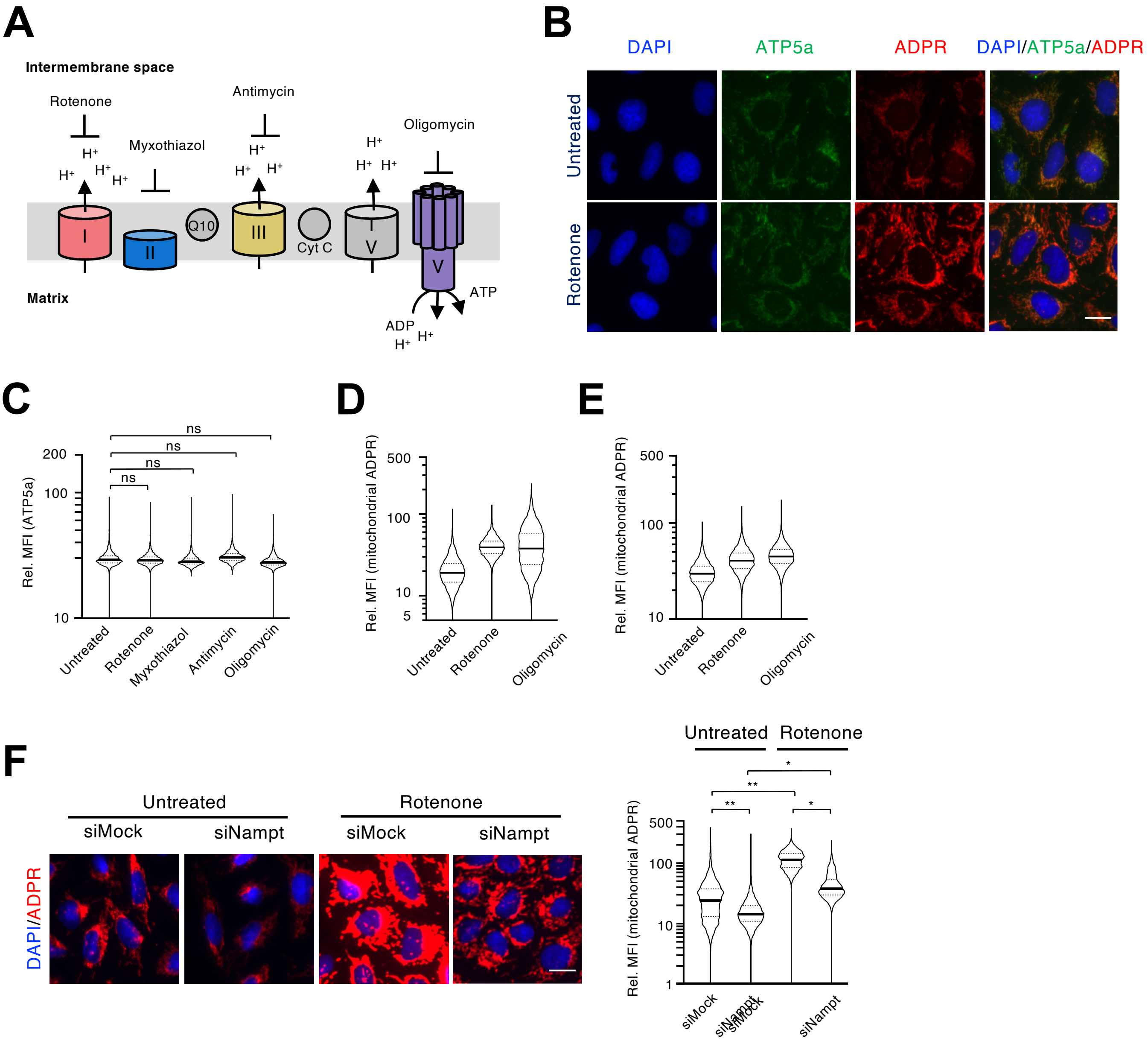

Suppl. Figure 3: Inhibition of respiration increases mitochondrial ADP-ribosylation, related to Figure 1

(A) Schematic overview of the respiratory chain, including the commonly used inhibitors Rotenone, Myxothiazol, Antimycin and Oligomycin. **(B)** Co-IF staining of ADP-ribosylation (anti-ADPr antibody; red) together with the ATP5a antibody (green) in untreated or Rotenone treated U2OS cells. **(C)** Quantification of the mitochondrial COXIV signal in U2OS cells after treatment with different respiratory chain inhibitors. **(D,E)** Quantification of mitochondrial ADP-ribosylation in A549 (**D**) and HeLa (**E**) cells after treatment with Rotenone or Oligomycin. **(F)** IF for ADP-ribosylation (anti-ADPr antibody; red) of U2OS subjected to 3 days of knockdown of NAMPT and subsequent Rotenone treatment. Representative pictures for each condition are shown on the left while the mean fluorescence intensity (MFI) of the mitochondrial ADP-ribosylation is shown on the right. The quantifications of all signals (mitochondrial and nuclear) were normalized as described in Fig.1. For the representation of all quantifications the signal for every event was normalized over the mean of the control/untreated sample, which was arbitrarily set to 30. The Y-axes of all violin plots are depicted as log 10 scales. Scale bars indicate 20 µm. For statistical analysis, a Student's t test was performed (n = 3-5; *p < 0.05; **p< 0.005; ***p < 0.0005).

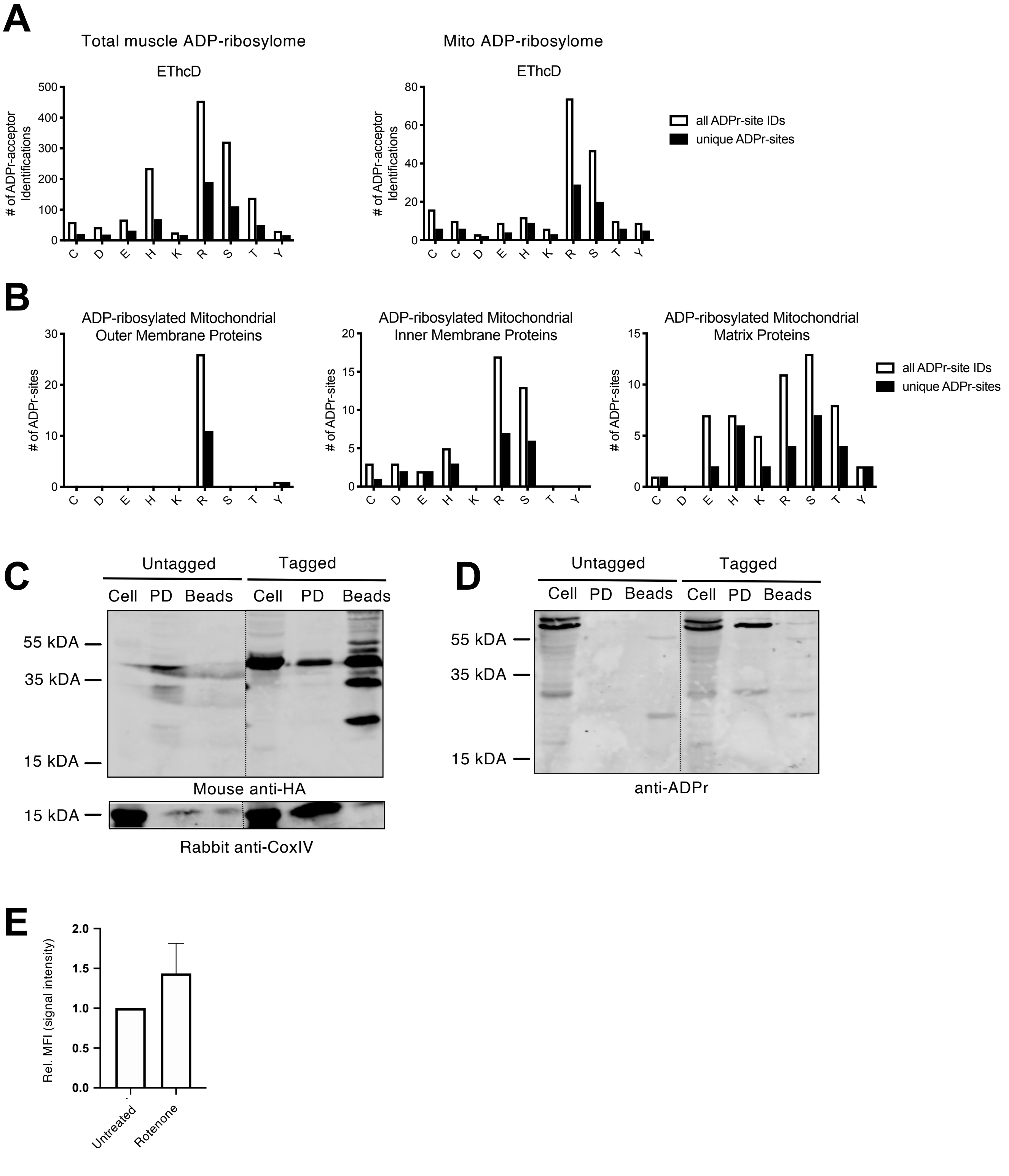

Suppl. Figure 4: Various mitochondrial inner- and outer membrane proteins are ADP-ribosylated, related to Figure 2

(A) Potential EThcD-defined amino acid acceptor site distributions identified in the total muscle ADP-ribosylome and the mitochondria-specific ADP-ribosylome. **(B)** Potential EThcD-defined amino acid acceptor site distributions on ADP-ribosylated mitochondrial proteins that localize to the outer membrane (left panel), the inner membrane (middle panel) or the matrix (right panel). **(C)** Pull down control of Fig. 2D: WB on mitochondrial lysates from U2OS cells stably transfected with HA-tagged OMP25 using an anti-HA- and an anti-COXIV antibody. **(D)** WB of mitochondrial lysates from U2OS cells stably transfected with HA-tagged OMP25 using the anti-ADPr antibody. **(E)** Quantification of the ADP-ribosylation signal intensity of Fig. 2E.

Suppl. Figure 5

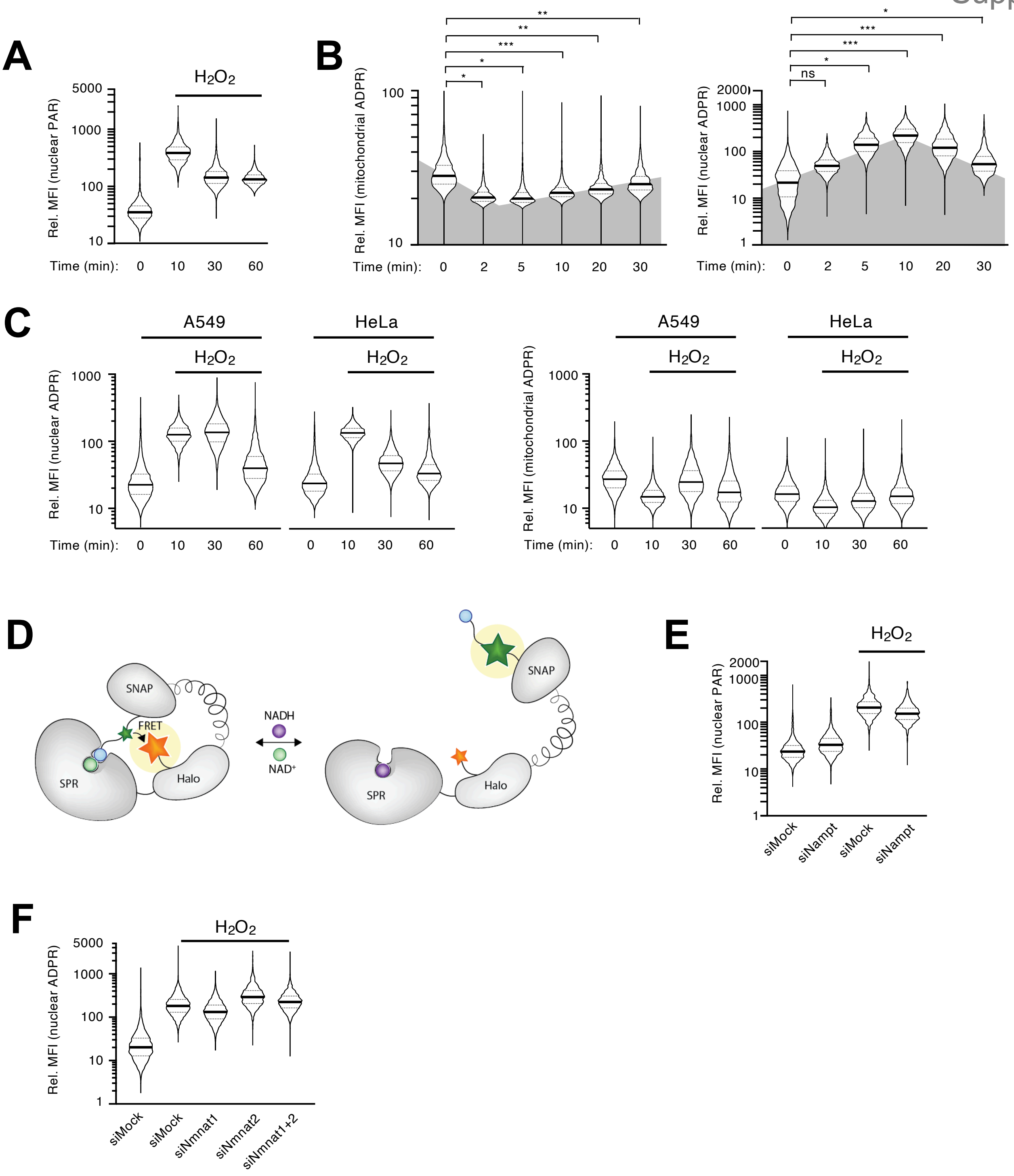

Suppl. Figure 5: Mitochondria-derived NAD^+ is crucial for the formation of nuclear PAR, related to Figure 3
(A,B) Quantification of the nuclear PAR **(A)** and nuclear as well as mitochondrial ADP-ribosylation **(B)** of the U2OS cells at various timepoints after H_2O_2 treatment. (**C**) Quantification of nuclear and mitochondrial ADP-ribosylation in A549 and HeLa cells at different time points after H_2O_2 treatment. **(D)** Visualization of the FRET-sensor constructs used for intracellular NAD^+ measurements. Scheme adapted from (Sallin et al., 2018). **(E)** U2OS cells were transfected with siRNA targeting NAMPT, treated with H_2O_2 and nuclear and nuclear ADP-ribosylation analyzed by IF using an anti-PAR antibody. **(F)** Quantification of nuclear ADP-ribosylation following 10 min of H_2O_2 treatment of U2OS after siRNA knockdown of NMNAT1, NMNAT2 or both together. The quantifications of all signals (mitochondrial and nuclear) were normalized as described in Fig. 1 and the Y-axes of all plots are depicted as log10 scale.

Suppl. Figure 6

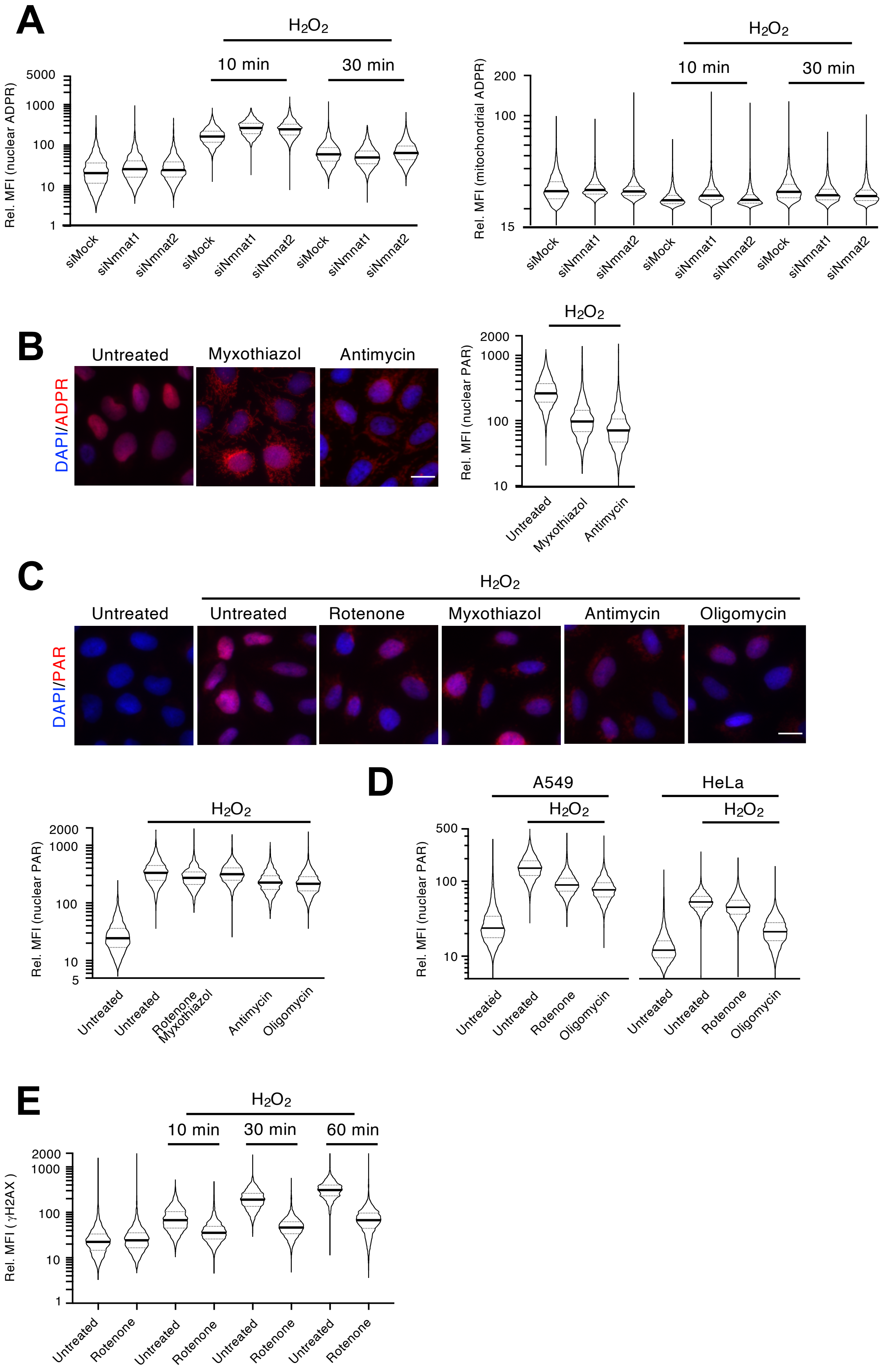

Suppl. Figure 6: Effect of NMNAT1/2 Knockdown on cellular ADP-ribosylation and Rotenone on H_2O_2 induced γH2AX formation, related to Figure 4

(A) U2OS cells were transfected with siRNA targeting NMNAT1 or 2 and nuclear (left panel) and mitochondrial (right panel) ADP-ribosylation was analyzed via IF and quantified at various timepoints following H_2O_2 treatment. **(B,C)** U2OS cells were pre-treated with either Rotenone, Myxothiazol, Antimycin or Oligomycin prior to a treatment with H_2O_2 and subsequent analysis of nuclear ADP-ribosylation via IF using an anti-ADPr antibody (**B**) or an anti-PAR antibody (**C**) . (**D**) A549 and HeLa cells were pre-treated with either Rotenone or Oligomycin before being treated with H_2O_2 and analyzed via IF. **(E)** U2OS cells were treated with H_2O_2 and Rotenone for the indicated time points and γH2AX levels were assessed via IF and quantified. The quantifications of all signals (mitochondrial and nuclear) were normalized as described in Fig. 1 and the Y-axes of all plots are depicted as log10 scale. Scale bars indicate 20 μm.

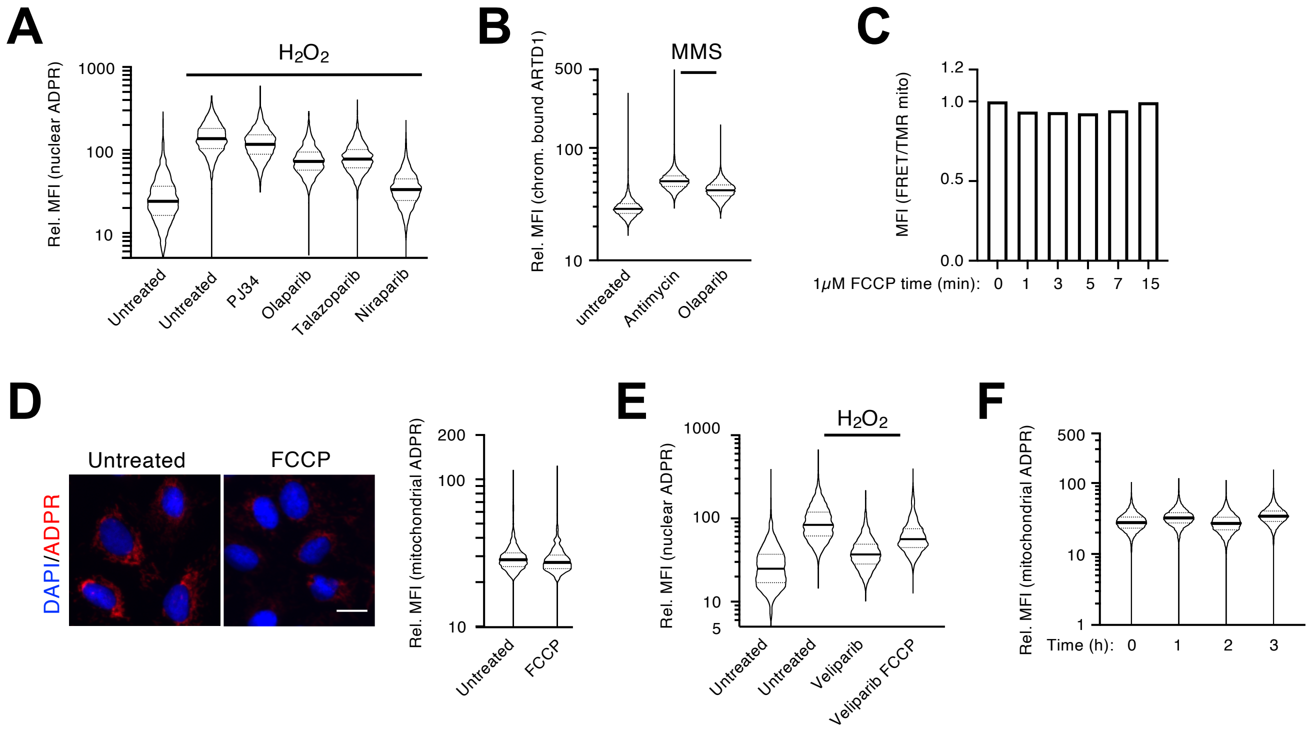

Suppl. Figure. 7: Effect of PARP inhibitors on nuclear and FCCP or PARG inhibitors on mitochondrial ADP-ribosylation, related to Figure 5

(A) U2OS cells were pre-treated for 1h with various PARP inhibitors and subsequently treated for 10 min with H_2O_2. **(B)** U2OS cells were co-treated with MMS and Antymicin for 2h and following pre-extraction, chromatin-bound ARTD1 was analyzed via IF. **(C)** Mitochondrial NAD^+ levels following FCCP treatment were assessed in stable U2OS Flp-In™ T-Rex™ cells expressing inducible NAD^+ sensors and analyzed via flow cytometry. Increased FRET ratios correspond to increased NAD^+ levels. **(D)** U2OS cells were treated with FCCP and mitochondrial ADP-ribosylation was analyzed via IF with the anti-ADPr antibody and quantified. (**E**) U2OS cells were pre-treated with Veliparib, then subjected to an FCCP pulse followed by H_2O_2 and analyzed via IF using an anti-ADPr antibody. (**F**) U2OS cell were treated with PARG inhibitor for the indicated time and mitochondrial ADP-ribosylation analyzed via IF using an anti-ADPr antibody. The quantifications of all signals (mitochondrial and nuclear) were normalized as described in Fig. 1 and the Y-axes of all plots are depicted as log10 scale. Scale bars indicate 20 µm.
