# Supplementary material for: Mitochondrial NAD+ Controls Nuclear ARTD1-Induced ADP-Ribosylation
Source: Mol Cell. 2021 Jan 21;81(2):340–354.e5. doi: 10.1016/j.molcel.2020.12.034 (PMC7837215; doi:10.1016/j.molcel.2020.12.034)
Supplement: Document S2. Article plus Supplemental Information [file mmc5.pdf]

Molecular Cell

Article

Mitochondrial NAD^+ Controls Nuclear ARTD1-Induced ADP-Ribosylation

Graphical Abstract

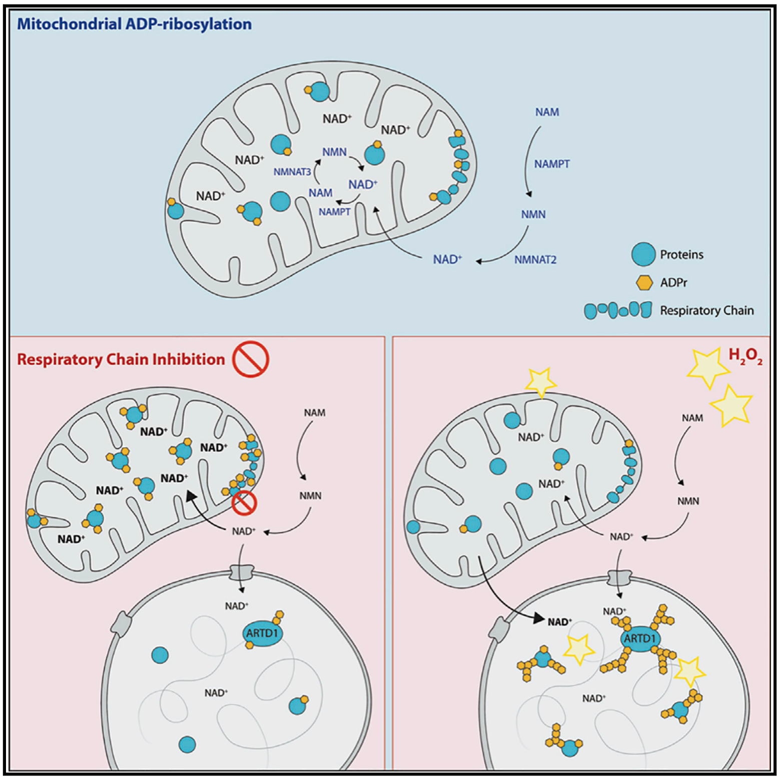

Authors

Ann-Katrin Hopp, Federico Teloni, Lavinia Bisceglie, ..., Matthias Altmeyer, Deena M. Leslie Pedrioli, Michael O. Hottiger

Correspondence

michael.hottiger@dmmd.uzh.ch

In Brief

Hopp et al. detect mitochondrial ADP-ribosylation and characterize its dependency on intracellular NAD^+ homeostasis. While respiratory chain inhibition increases mitochondrial ADP-ribosylation, hydrogen peroxide treatment reduces mitochondrial ADP-ribosylation and reciprocally induces nuclear ADP-ribosylation. This dynamic and reversable process is dependent on a NAD^+-dependent mitochondrial-nuclear crosstalk.

Highlights

- Mitochondrial ADP-ribosylation was identified by different methods
- Mitochondrial ADP-ribosylation reversibly increased after respiratory chain inhibition
- H_2O_2 treatment induces nuclear and reduces mitochondrial ADP-ribosylation
- Elevated mitochondrial ADP-ribosylation dampened MMS-induced ARTD1 chromatin retention

Hopp et al., 2021, Molecular Cell *81*, 340–354
January 21, 2021 © 2021 The Authors. Published by Elsevier Inc.
https://doi.org/10.1016/j.molcel.2020.12.034

CellPress

CellPress
OPEN ACCESS

Molecular Cell

Article

Mitochondrial NAD^+ Controls Nuclear ARTD1-Induced ADP-Ribosylation

Ann-Katrin Hopp,[1,2] Federico Teloni,[1,2] Lavinia Bisceglie,[1,2] Corentin Gondrand,[3,8] Fabio Raith,[3,4,8] Kathrin Nowak,[1,2] Lukas Muskalla,[1,5] Anna Howald,[1] Patrick G.A. Pedrioli,[6,7] Kai Johnsson,[3] Matthias Altmeyer,[1] Deena M. Leslie Pedrioli,[1] and Michael O. Hottiger[1,9,*]
[1]Department of Molecular Mechanisms of Disease (DMMD), University of Zurich, 8057 Zurich, Switzerland
[2]Life Science Zurich Graduate School, Molecular Life Science Ph.D. Program, University of Zurich, 8057 Zurich, Switzerland
[3]Department of Chemical Biology, Max Planck Institute for Medical Research, 69120 Heidelberg, Germany
[4]Faculty of Chemistry and Earth Sciences, University of Heidelberg, 69120 Heidelberg, Germany
[5]Life Science Zurich Graduate School, Cancer Biology Ph.D. Program, University of Zurich, 8057 Zurich
[6]Department of Biology, Institute of Molecular Systems Biology, ETH Zurich, 8093 Zurich, Switzerland
[7]PHRT-CPAC, ETH Zurich, 8093 Zurich, Switzerland
[8]These authors contributed equally
[9]Lead Contact
*Correspondence: michael.hottiger@dmmd.uzh.ch
https://doi.org/10.1016/j.molcel.2020.12.034

SUMMARY

In addition to its role as an electron transporter, mitochondrial nicotinamide adenine dinucleotide (NAD^+) is an important co-factor for enzymatic reactions, including ADP-ribosylation. Although mitochondria harbor the most intra-cellular NAD^+, mitochondrial ADP-ribosylation remains poorly understood. Here we provide evidence for mitochondrial ADP-ribosylation, which was identified using various methodologies including immunofluorescence, western blot, and mass spectrometry. We show that mitochondrial ADP-ribosylation reversibly increases in response to respiratory chain inhibition. Conversely, H_2O_2-induced oxidative stress reciprocally induces nuclear and reduces mitochondrial ADP-ribosylation. Elevated mitochondrial ADP-ribosylation, in turn, dampens H_2O_2-triggered nuclear ADP-ribosylation and increases MMS-induced ARTD1 chromatin retention. Interestingly, co-treatment of cells with the mitochondrial uncoupler FCCP decreases PARP inhibitor efficacy. Together, our results suggest that mitochondrial ADP-ribosylation is a dynamic cellular process that impacts nuclear ADP-ribosylation and provide evidence for a NAD^+-mediated mitochondrial-nuclear crosstalk.

INTRODUCTION

Nicotinamide adenine dinucleotide (NAD^+) is an essential small molecule that functions not only as an important redox equivalent but also as a co-factor for various enzymes (Chiarugi et al., 2012; Katsyuba and Auwerx, 2017). In mammals, NAD^+ can be synthesized *de novo* from tryptophan or via the Preiss-Handler pathway from nicotinic acid (NA). Alternatively, NAD^+ can be generated via the salvage pathway from nicotinamide or by the nicotinamide ribose kinase pathway (Bogan and Brenner, 2008; Strømland et al., 2019). In-depth analysis revealed that the vast majority of immortalized cells depend entirely on the salvage pathway (Liu et al., 2018) and require two types of enzymes: nicotinamide phosphoribosyltransferase (NAMPT) and nicotinamide mononucleotide adenylyltransferases (NMNATs). While the rate-limiting step of the NAD^+ salvage pathway is the NAMPT-mediated conversion of NAM to NMN (Revollo et al., 2004), NMNATs convert NMN to NAD^+.

Since NAD^+ is considered to be membrane impermeable (Stein and Imai, 2012), NAD^+-dependent signaling pathways are compartmentalized within the cell. The NAD^+ concentration is high within mitochondria (~400 μM, 40%–70% of the total cellular NAD^+ pool) (Alano et al., 2007; Di Lisa et al., 2001), intermediate in the nucleus and cytosol (~100 μM), and low (< 1 μM) in extracellular spaces (Cambronne et al., 2016; Sallin et al., 2018). These concentrations also vary considerably depending on the cell type, metabolic condition, stress, and redox status (Koch-Nolte et al., 2011). Cytoplasmic and nuclear NAD^+/NADH ratios are typically maintained between 60 and 700 in eukaryotes, depending on cell type; the mitochondrial ratio is around 7–8 (Williamson et al., 1967; Zhang et al., 2002). In mitochondria, the maintenance of NAD^+ levels and NAD^+/NADH ratios are crucial for mitochondrial function, homeostasis, and ATP production. The mitochondrial respiratory chain (complexes I–V) is the major site of ATP production in eukaryotes. In addition, electron reduction of O_2 during oxidative phosphorylation generates water and reactive oxygen species (ROS) that are subsequently converted to hydrogen peroxide (H_2O_2). Mitochondria-derived ROS (mROS) were initially thought to exclusively cause cellular damage, but we now understand that mROS are

This is an open access article under the CC BY license (http://creativecommons.org/licenses/by/4.0/).

CellPress
OPEN ACCESS

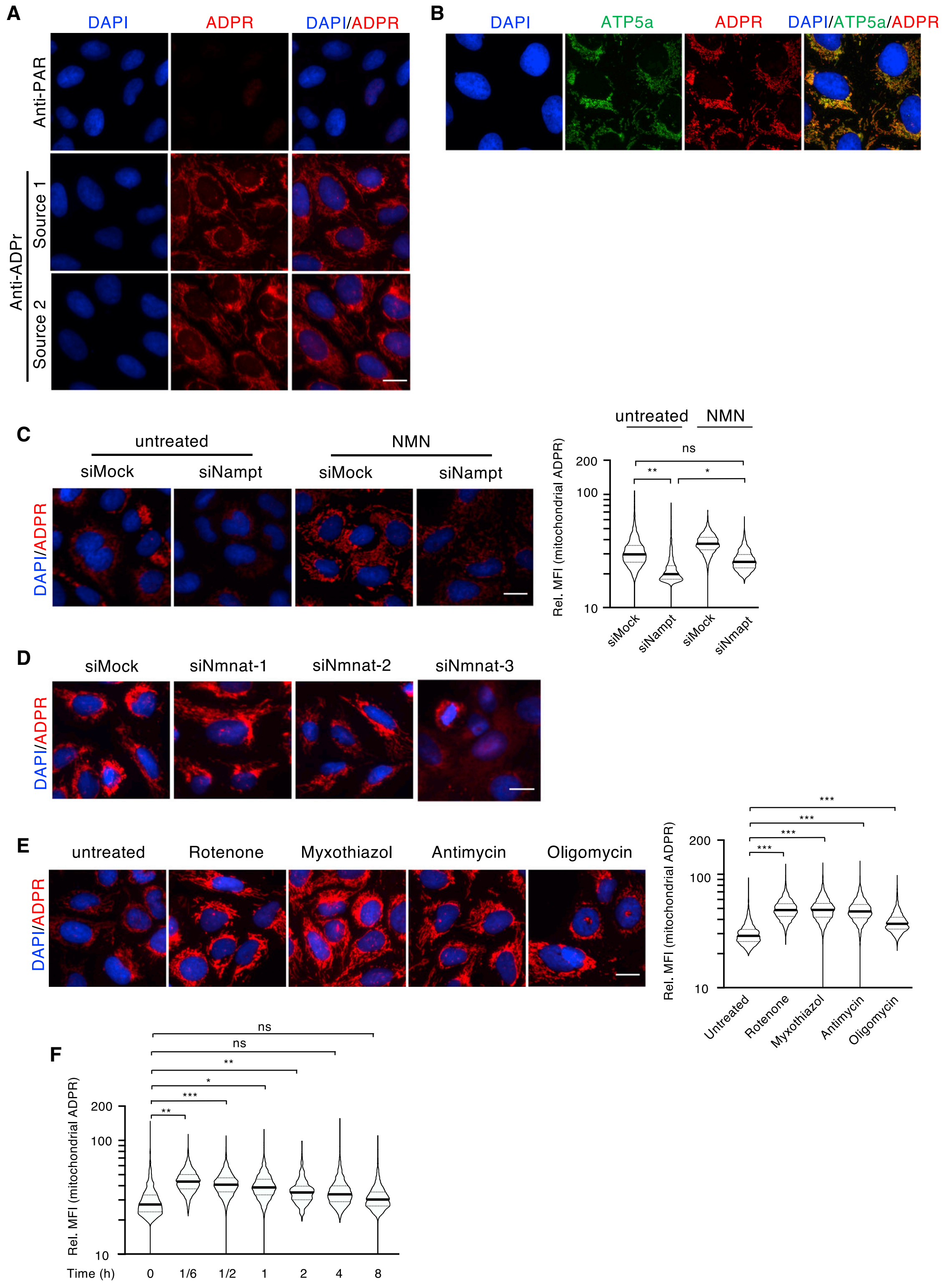

Figure 1. Mitochondrial ADP-Ribosylation Adapts in Response to Metabolic Changes

(A) Immunofluorescence (IF) staining of ADP-ribosylation (red) in U2OS cells using either an anti-PAR (upper panel) or two different pan-anti-ADPr antibodies (lower panels).

(B) Co-localization of ADP-ribosylation (red) and ATP5a (green).

(legend continued on next page)

important signaling molecules that regulate several physiological processes (Sena and Chandel, 2012).

NAD^+ is additionally consumed by multiple families of signaling enzymes, including NAD kinase (Li et al., 2018), cyclic ADP-ribose hydrolases (CD38/ CD157) (Horenstein et al., 2009), sirtuins (SIRTs) (Haigis and Sinclair, 2010), and the family of NAD^+-consuming ADP-ribosyltransferases (ARTs). ARTs catalyze the post-translational attachment of one (mono-ADP-ribosylation [MAR]; MARylation) or several (poly-ADP-ribosylation [PAR]; PARylation) moieties of ADP-ribose (ADPr) from NAD^+ onto specific acceptor proteins (Hottiger, 2015). Protein ADP-ribosylation has been implicated in a plethora of different cellular processes, including genomic stability, transcriptional control, energy metabolism, and cell death (Hottiger, 2015; Kraus, 2015). Although intracellular NAD^+ levels decrease by only 5%–10% upon moderate PAR synthesis, severe stress-induced PAR synthesis results in near-complete depletion of the cellular NAD^+ pool, which ultimately leads to necrotic cell death (Ha and Snyder, 1999). A recent study demonstrated that cytoplasmic/nuclear-specific NAD^+ biosynthesis is a key mediator of ADP-ribosyltransferase diphtheria toxin-like 1 (ARTD1)/poly-(ADP-ribosyl)polymerase 1 (PARP1)-regulated transcription during adipocyte differentiation; thus connecting the cellular metabolic state with adipogenesis (Ryu et al., 2018). Whether mitochondria, with their high NAD^+ concentrations, also contribute to NAD^+-regulated processes in other cellular compartments is currently not known. Here, we investigated mitochondrial ADP-ribosylation and analyzed its regulation, as well as function, under different stress conditions.

RESULTS

In Situ Detection of Mitochondrial Mono-ADP-Ribosylation

MARylation has been described to regulate several important biological processes (Lüscher et al., 2018). Detection of cellular MARylation has remained challenging due to the fact that common anti-ADP-ribosylation antibodies preferentially bind to long PAR chains (Kanai, 2016). While synthesis of chemically ADP-ribosylated peptides for antibody generation has remained challenging (Voorneveld et al., 2018), amine-linked ADP-ribose-modified proteins have been used to successfully generate polyclonal antibodies that recognize MARylated proteins (Meyer and Hilz, 1986). Using MARylated peptides as antigens, we developed a polyclonal ADPr-specific antibody that recognizes MARylated peptides in ELISA assays in a manner comparable to a new commercially available pan-ADPr antibody (Figure S1A; source 1 and source 2, respectively). Our anti-ADPr antibody (source 1) also detected MARylated proteins (e.g., purified ARTD10, ARTD8, or immunoprecipitated proteins; Xu et al., 2019) as well as PARylated proteins (e.g., purified ARTD1) by western blot (WB) (Figure S1B). In addition, dot blot analyses with PAR, ADPr, and different adenosine moities demonstrated that this antibody specifically recognized single ADPr moieties and isolated PAR, but not AMP, ADP, ATP, nor GTP (Figure S1B). Together, these findings indicate that the antibody is ADPr-specific and does not cross-react with other related metabolites *in vitro*. Interestingly, when used in immunofluorescence (IF), the anti-ADPr antibody detected a strong extranuclear signal in untreated U2OS cells that was not observed with the anti-PAR antibody (Figure 1A). Importantly, this extranuclear ADP-ribosylation signal was also observed by IF when cells were stained with a commercially available pan anti-ADPr antibody, as well as with an engineered Af1521-macrodomain fused to the mouse Fc fragment (Nowak et al., 2020), capable of binding MARylated and PARylated proteins (Figure S1C). Moreover, the signal observed with the anti-ADPr antibody was also observed in other human (e.g., HeLa, A549, and IMR90) and murine (e.g., C2C12 myoblasts) cell lines (Figure S1D), suggesting that the signal was not cell-type or species specific. When tested in combination with different organelle markers, we observed a distinct overlap between the ADP-ribosylation signal and a mitochondrial marker (subunit 5a of the mitochondrial ATP synthase [ATP5a]), which suggested that mitochondria contain ADP-ribosylated proteins (Figure 1B). To confirm that this mitochondrial signal in cells was indeed a post-translational protein modification, cells were treated for 30 min with cycloheximide before fixation and IF staining. Treatment with cycloheximide strongly reduced the intensity of the observed IF signal (Figure S1E), suggesting that the signal was protein-synthesis dependent and excluding that the antibody recognized free or precipitated metabolites. Since 30 min were sufficient to strongly reduce the signal, this finding further indicates that mitochondrial ADP-ribosylation is dynamic. To further exclude that the detected mitochondrial signal is catalyzed by SelO (a mitochondrial pseudokinase), which has recently been shown to AMPylate a subset of mitochondrial redox proteins (Sreelatha et al., 2018), we knocked down SelO with two independent siRNAs (Figure S1F). This knockdown did not affect the mitochondrial signal (Figure S1G), further strengthening our conclusion that it is indeed ADP-ribosylation.

Mitochondrial ADP-Ribosylation Depends on NAMPT and NMNAT3

As most transformed cells rely on the salvage pathway for the synthesis of NAD^+, knockdown of NAMPT should reduce mitochondrial ADP-ribosylation. We, thus, knocked down NAMPT in U2OS cells (Figure S2A) and quantified ADP-ribosylation signal intensities in single cells via quantitative image-based cytometry (QIBC) (Altmeyer et al., 2013; Toledo et al., 2013) using

(C) Influence of NAMPT knockdown and NMN supplementation on mitochondrial ADP-ribosylation. Left panel: representative IF images of the staining with an anti-ADPr antibody (red); right panel: quantification of the relative mean fluorescence intensity (Rel. MFI) of the mitochondrial ADP-ribosylation signal.
(D) Influence of NMNAT1, 2, or 3 on mitochondrial ADP-ribosylation.
(E) IF staining for ADP-ribosylation after treatment with inhibitors for the respiratory chain complexes and for the ATP synthase.
(F) Pulse-chase treatment of U2OS cells with rotenone for 10 min and recovery for the indicated time.
For representation of all quantifications, the signals for every event were normalized over the mean of the control/untreated, which was arbitrarily set to 30. The y axes of all violin plots are depicted as log10 scales. Scale bars indicate 20 μm. For statistical analysis, a Student's t test was performed (n = 3–5; *p < 0.05; **p < 0.005; ***p < 0.0005).

CellPress
OPEN ACCESS

detection masks that corresponded to mitochondria (i.e., ATP5a or CoxIV co-staining) or nuclei (i.e., DAPI signal) (Figure S2B). Mitochondrial ADP-ribosylation signals were significantly reduced following NAMPT knockdown or NAMPT inhibition by FK866 (Figure 1C; Figure S2C), demonstrating that mitochondrial ADP-ribosylation is dependent on NAD^+. Importantly, the signal intensities of CoxIV remained unchanged, confirming that knockdown or inhibition of NAMPT did not alter the cellular mitochondrial load (Figures S2D and S2E). Complementing cells with NMN, which unlike NAD^+ can be transported into cells, should compensate for NAMPT loss of function by directly stimulating NAD^+ synthesis via the NMNATs (Grozio et al., 2019). Indeed, addition of NMN to the media after NAMPT knockdown increased the basal signals observed in siMock transfected cells and to a large extent restored mitochondrial ADP-ribosylation signals (Figure 1C). To confirm that mitochondrial NAD^+ is required for mitochondrial ADP-ribosylation, we reduced the expression of the three NMNAT enzymes (NMNAT1, NMNAT2, and NMNAT3; Figure S2A) and assessed mitochondrial ADP-ribosylation via IF using the anti-ADPr antibody. Knockdown of NMNAT1, NMNAT2, or both together had no effect on mitochondrial ADP-ribosylation (Figure 1D; Figure S2F). Interestingly, knockdown of mitochondrial NMNAT3 altered the well-defined mitochondrial signal to a more diffuse signal. Together, these data indicate that the ADP-ribosylation signals observed in mitochondria are dependent on cellular NAD^+ availability and that mitochondrial NAD^+ concentrations strongly contribute to mitochondrial ADP-ribosylation under basal conditions.

Inhibition of the Respiratory Chain Increases Mitochondrial Anti-ADP-Ribosylation Signals

Mitochondrial NAD^+ is best known for its role in transferring electrons from its reduced form, NADH, via the citric acid cycle to the respiratory chain, which promotes oxidative phosphorylation (Smeitink et al., 2006). Inhibition of the respiratory chain leads to electron leakage, depolarization of the inner mitochondrial membrane, and mROS production (Li et al., 2003; Selivanov et al., 2008). Treatment of U2OS cells for 30 min with complex I, II, or III respiratory chain inhibitors (i.e., rotenone, myxothiazol, or antimycin; Figure S3A) significantly induced mitochondrial ADP-ribosylation without altering ATP5a protein levels (Figure 1E; Figures S3B and S3C). Together, these data indicate that mitochondrial ADP-ribosylation correlates with cellular respiratory dysfunction. A comparable increase in mitochondrial ADP-ribosylation following rotenone treatment was also observed by IF in other human cell lines (i.e., A549 or HeLa). Similar results were also observed when the IFs were performed with a commercially available pan-ADP-ribose antibody or the engineered Af1521 macrodomain-FC fusion protein (Figures S1C, S3D, and S3E). Moreover, oligomycin treatment, which inhibits the mitochondrial F0F1 ATP synthase (Symersky et al., 2012), also resulted in increased mitochondrial ADP-ribosylation in different cell lines tested (Figure 1E; Figures S3D and S3E). The rotenone-induced mitochondrial ADP-ribosylation signals remained NAMPT dependent (Figure S3F) and were reversible after removal of rotenone (Figure 1F), further supporting our hypothesis that the changes observed are very dynamic and mediated by ADP-ribosylating and/or NAD^+-consuming enzymes.

Identification of the Mitochondrial ADP-Ribosylome in Muscle Cells

The mitochondrial ADP-ribosylation observed by IF prompted us to identify the proteins that are ADP-ribosylated in U2OS cells (i.e., the mitochondrial ADP-ribosylome). Analyses of the baseline U2OS ADP-ribosylome using our established ADP-ribosylome proteomic workflow (Martello et al., 2016) led to the identification of 97 unique ADP-ribosylated proteins (Table S1). We cross-referenced these ADP-ribosylated proteins with the Human MitoCarta2.0 inventory (Calvo et al., 2016) and identified six ADP-ribosylated mitochondrial proteins: ACOT1, ACOT2, ATP5MF, TOMM70, DISC1, and GAPDH. Similar to previous studies (Buch-Larsen et al., 2020; Larsen et al., 2018), the number of ADP-ribosylated mitochondrial proteins relative to the full ADP-ribosylome identified was rather low. We, therefore, identified the ADP-ribosylome of murine muscle tissues, which have higher mitochondrial loads than cultured cells. In murine muscle, we identified 518 unique ADP-ribosylated peptides that mapped to 194 unique proteins (Figure 2A; Table S2). Comparison of these data to the Mouse MitoCarta2.0 (Calvo et al., 2016) and the UniProt subcellular protein localization annotations (https://www.uniprot.org) inventory led to the identification of 49 (26% of the total ADP-ribosylome) ADP-ribosylated mitochondrial proteins (Figure 2A; Table S3). For the majority of these ADP-ribosylated proteins, only one particular modified region was observed (Figure 2B; Table S3).

A detailed analysis of the modified mitochondrial proteins with respect to mitochondrial topology revealed that these proteins localize throughout the mitochondria and, based on MS-PSM counts, suggested that proteins localizing to the inner membrane of the mitochondria and/or mitochondrial matrix potentially represent the most heavily ADP-ribosylated mitochondrial proteins (Figure 2C). Strikingly, the ADP-ribosylated proteins that we identified regulate important mitochondrial functions (Figure 2C). In agreement with one of our previous studies (Leutert et al., 2018), these data also confirm that arginine is a prominent ADPr-acceptor amino acid in muscle tissue (Figure S4A). Further analysis of the mitochondrial ADP-ribosylome with respect to ADPr-acceptor sites suggested that the ADPr-acceptor amino acids may vary depending on where the modified proteins localize within the mitochondria (Figure S4B).

Based on the successful identification of ADP-ribosylated mitochondrial proteins in muscle tissues, we aimed to confirm that the mitochondrial ADP-ribosylation signal observed in U2OS cells indeed reflects protein ADP-ribosylation. We therefore isolated mitochondria using a recently developed fast mitochondrial enrichment technique (Chen et al., 2016). WB analysis of the resulting mitochondrial lysates with the anti-ADPr antibody led to the detection of several protein bands in whole-cell lysates and in the mitochondrial pull-down (PD) fraction (Figure S4D). Validating the IF data, WB analysis of mitochondria isolated from untreated and rotenone-treated U2OS cells with the anti-ADPr antibody revealed an increased ADP-ribosylation signal following rotenone treatment (Figure 2D; Figure S4E). Using a pan anti-ADPr antibody, the difference in overall mitochondrial protein ADP-ribosylation of

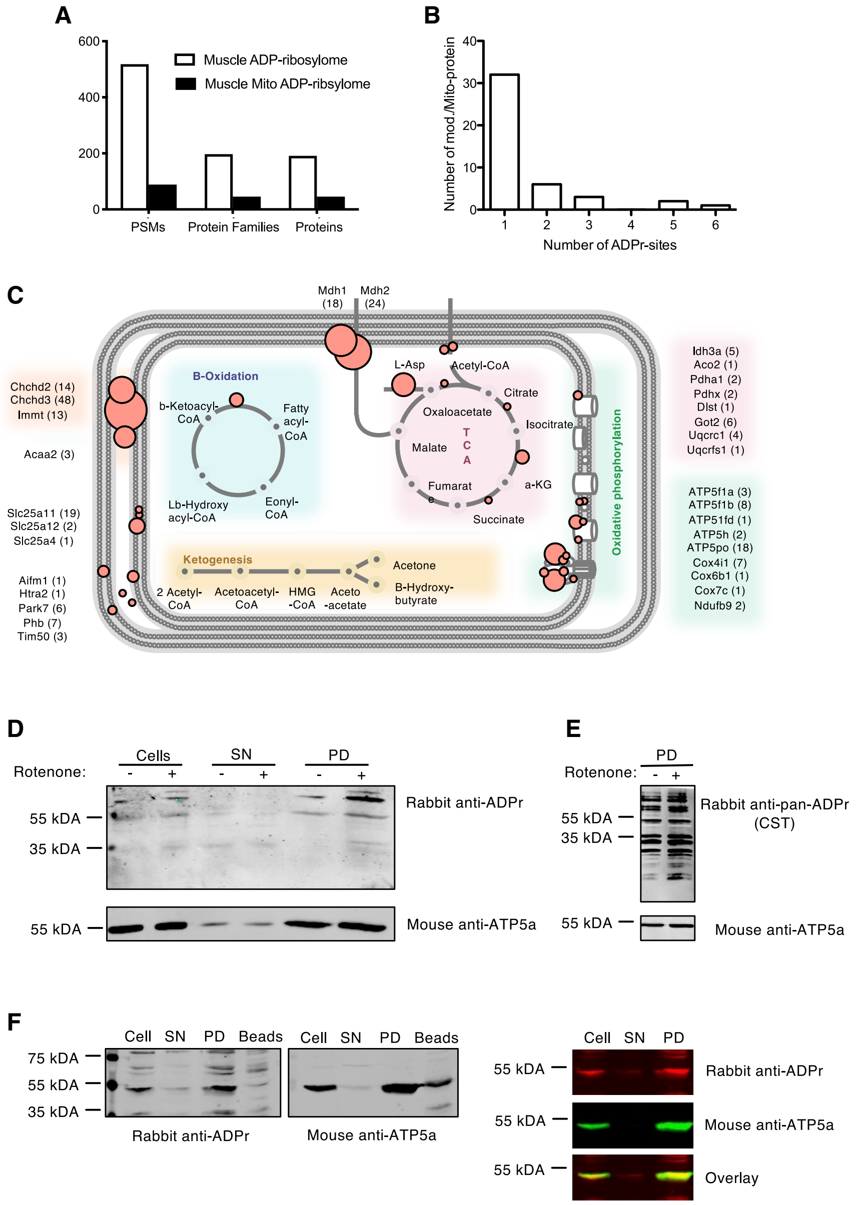

Figure 2. Identification of Mitochondrial ADP-Ribosylated Proteins by Mass Spectrometry

(A) Number of peptide spectral matches (PSMs), protein families, and proteins identified to be ADP-ribosylated in mouse muscle tissue (white, total proteins; black, mitochondrial proteins).

(B) Frequency of ADP-ribosylation regions identified on mitochondria-localized proteins.

(C) Schematic overview of the distribution of protein ADP-ribosylation detected in mitochondria from murine muscle. ADP-ribosylation is indicated using a red circle. Each circle represents an ADP-ribsosylated protein or subunit; the size of each circle depicts the number of times the modified peptide/protein was identified by MS, and spectral counts for each protein are indicated in parentheses.

(D) Anti-ADPr antibody stained western blot of whole-cell and mitochondrial lysates isolated from untreated and rotenone-treated U2OS cells stably transfected with HA-tagged OMP25.

(E) WB on mitochondrial lysates from untreated and H_2O_2-treated cells stained with a commercial pan-ADPr antibody.

(F) WB stained for ADP-ribosylation and ATP5a on mitochondrial lysates isolated from U2OS cells stably transfected with HA-tagged OMP25 (SN, supernatant; PD, pull-down).

lysates derived from untreated or rotenone-treated cells was increased by 30% (Figure 2E; Figure S4F). These WB results (Figures 2D and 2E) recapitulate the IF data presented above (Figure 1E) and further support that the observed ADP-ribosylation signals are indeed protein bound. Interestingly, we also observed an overlap of the ATP5a and ADP-ribosylation signals in the cell lysates and mitochondria-enriched fractions from U2OS cells (Figure 2F). These data, together with our U2OS and mouse muscle ADP-ribosylome MS data (Tables S2 and S3), indicate that ATP5a is ADP-ribosylated in both mouse and human cells.

Strong Induction of Nuclear ADP-Ribosylation Reduces Mitochondrial ADP-Ribosylation in a Reversible Manner

H_2O_2 treatment induces strong nuclear PARylation in a time- and IP3/calcium-dependent manner (Andersson et al., 2016). To investigate whether mitochondrial ADP-ribosylation signals were also affected by H_2O_2, cells were treated with H_2O_2 for 0, 10, 30, and 60 min, and cellular ADP-ribosylation was monitored by IF using either our own anti-ADPr or an anti-PAR antibody. In agreement with our previous study (Andersson et al., 2016), strong nuclear PARylation was detected with the anti-PAR antibody 10 min after H_2O_2 treatment and dissipated after 60 min (Figure 3A upper panel, quantification in Figure S5A). The anti-ADPr antibody also detected dynamic H_2O_2-induced nuclear ADP-ribosylation similar to that observed with the anti-PAR antibody (Figure 3A lower panel, quantification right panel). Interestingly, basal mitochondrial ADP-ribosylation was significantly reduced after 10 min of H_2O_2 treatment and recovered after 60 min (Figure 3A lower panel, quantification right panels). To analyze the reduction in mitochondrial ADP-ribosylation following H_2O_2 treatment more thoroughly, a detailed time course that included 2, 5, and 20 min time points was performed, and changes in both mitochondrial and nuclear ADP-ribosylation were investigated by IF (Figure S5B). Intriguingly, while nuclear ADP-ribosylation peaked 10 min post-H_2O_2 treatment, the reduction in mitochondrial ADP-ribosylation was most pronounced between 2 and 5 min post-H_2O_2 treatment. Similar ADP-ribosylation dynamics were also observed in A549 and HeLa cells (Figure S5C). Taken together, these data reveal dynamic and

CellPress
OPEN ACCESS

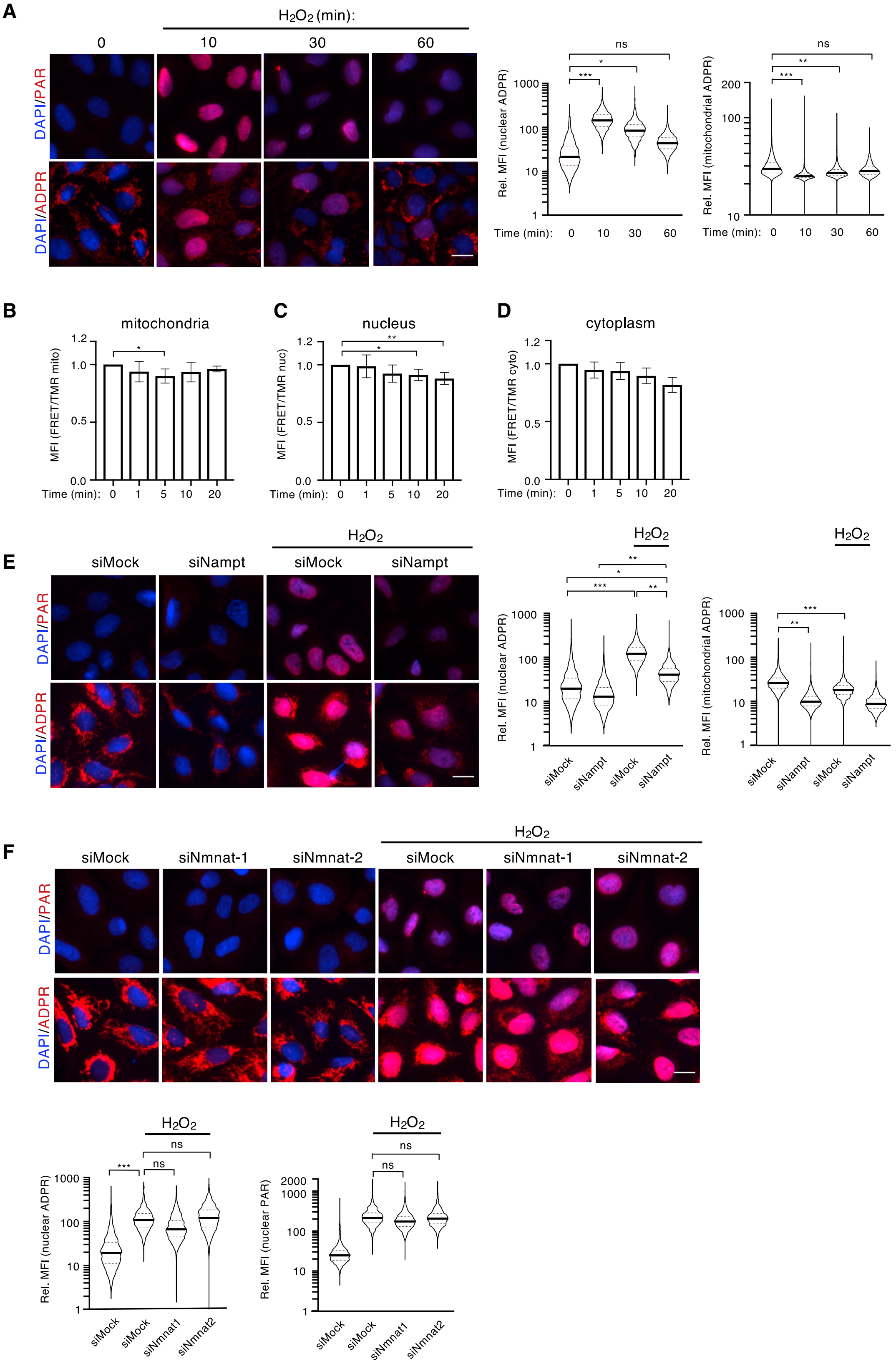

(legend on next page)

inversely correlated mitochondrial and nuclear ADP-ribosylation changes.

The inverse correlation observed between mitochondrial and nuclear ADP-ribosylation suggested that the mitochondrial NAD^+ pool could serve as a source for H_2O_2-induced nuclear ADP-ribosylation. In fact, extensive nuclear ADP-ribosylation generated after severe genotoxic stress consumes large amounts of NAD^+ (Schraufstatter et al., 1986). To investigate NAD^+ concentration changes in different subcellular compartments after H_2O_2 treatment, stable U2OS Flp-In T-Rex cells expressing inducible NAD^+ sensors that localize to either the nucleus, cytoplasm, or mitochondria were used (Figure S5D; Sallin et al., 2018). The three cell lines were treated with H_2O_2, and the NAD^+ sensor FRET ratios assessed at various time points after treatment (Figures 3B–3D). While mitochondrial NAD^+ levels dropped very quickly in response to H_2O_2 treatment (within 1–5 min) and subsequently recovered (Figure 3B), nuclear and cytoplasmic NAD^+ levels experienced a continuous drop over time starting only after 5 min (Figures 3C and 3D). Intriguingly, the changes in mitochondrial NAD^+ showed the same dynamics as mitochondrial ADP-ribosylation levels, suggesting that the drop in mitochondrial ADP-ribosylation after H_2O_2 administration was a consequence of decreasing mitochondrial NAD^+. Moreover, given that neither cytoplasmic nor nuclear NAD^+ levels increased after H_2O_2 treatment, it appears as though mitochondria-derived NAD^+ is immediately consumed by nuclear ARTD1.

H_2O_2-Induced Nuclear ADP-Ribosylation Requires NAMPT

The inverse correlation observed between mitochondrial and nuclear ADP-ribosylation and the measured NAD^+ fluctuations suggested that mitochondrial NAD^+ pools could be used to generate nuclear ADP-ribosylation in response to H_2O_2. To determine whether the NAD^+ salvage pathway contributes to H_2O_2-induced nuclear ADP-ribosylation, NAMPT was depleted in U2OS cells prior to 10 min H_2O_2 treatment. Nuclear ADP-ribosylation was then quantified using both the anti-ADPr and the anti-PAR antibodies (Figure 3E, upper panel and quantification, Figure S5E). Depletion of NAMPT strongly dampened both basal mitochondrial ADP-ribosylation and H_2O_2-induced nuclear ADP-ribosylation, indicating that the NAD^+ salvage pathway is required for both nuclear and mitochondrial ADP-ribosylation (Figure 3E lower panel and quantification right panels).

To further support our hypothesis that H_2O_2-induced nuclear PARylation is mediated via NAD^+ release from the mitochondria rather than continuous nuclear and/or cytoplasmic synthesis, the contribution of the nuclear and the cytoplasmic NMNATs (NMNAT1 and 2, respectively) to mitochondrial and nuclear ADP-ribosylation following H_2O_2 treatment was tested. To this end, NMNAT1 and 2 were knocked down either alone or in combination, and cells were treated with H_2O_2 to induce nuclear ADP-ribosylation. Quantification of mitochondrial and nuclear anti-PAR or anti-ADP-ribosylation signals demonstrated that H_2O_2-induced nuclear ADP-ribosylation signals were comparable between all conditions (Figure 3F; Figure S5F). Taken together, these data indicate that NMNAT1 and NMNAT2 do not contribute to H_2O_2-induced nuclear ADP-ribosylation at the time point measured here and suggest that pre-existing mitochondrial NAD^+ is likely released upon H_2O_2 treatment and consumed within the nucleus.

Knockdown of NMNAT3 and the NMN-Transporter SLC12A8 Extends Nuclear ADP-Ribosylation after H_2O_2 Treatment

Our finding that mitochondrial NAD^+ levels were restored within 10–15 min of H_2O_2 treatment suggests that NAM, generated following PARylation, might be directly recycled to compensate for the loss of cellular NAD^+. In addition, our observation that nuclear and cytoplasmic NAD^+ levels continued to decrease even 20 min post-treatment indicates that cells first restore the mitochondrial NAD^+ pool. H_2O_2-induced ADP-ribosylation releases considerable amounts of NAM that can be converted to NMN by NAMPT. NMN can either be converted to NAD^+ in the nucleus/cytoplasm or be imported into mitochondria. Once imported, NMN would then be converted to NAD^+ via the local NMNAT3, thus restoring both mitochondrial NAD^+ and ADP-ribosylation levels (Figure 4A). To assess to what extent cellular NMN import contributes to mitochondrial NAD^+ re-establishment after H_2O_2 treatment, the cellular NMN transporter SLC12A8 was knocked down in U2OS cells (Grozio et al., 2019). Quantification of nuclear and mitochondrial ADP-ribosylation levels upon SLC12A8 knockdown and at several time points following treatment revealed that the dynamics of nuclear PAR formation remained high at the 30 min time point compared to control cells (Figures 4B and 4C). These findings suggest that the extended H_2O_2-induced PAR formation is dependent on

Figure 3. Mitochondria-Derived NAD^+ Is Crucial for the Formation of Nuclear PAR

(A) U2OS cells were treated with H_2O_2 for the indicated amount of time. Nuclear and mitochondrial ADP-ribosylation were assessed by IF using an anti-PAR antibody (upper pictures) and an anti-ADPr antibody (lower pictures, both red). Representative pictures for each condition are shown on the left; the quantification of nuclear (left) and mitochondrial (right) IF signals detected with the anti-ADPr antibody is depicted on the right.

(B–D) Stable U2OS Flp-In T-Rex cells expressing inducible NAD^+ sensors targeted to the mitochondria (B), the nucleus (C), or the cytoplasm (D) were subjected to H_2O_2 treatment, and NAD^+ levels in each compartment were assessed at various time points via flow cytometry. Increased FRET ratios correspond to increased NAD^+ levels. FRET ratios of three independent experiments including square deviation are shown.

(E) U2OS cells were transfected with siRNA targeting NAMPT and treated with H_2O_2, and nuclear and mitochondrial ADP-ribosylation was analyzed by IF using an anti-PAR antibody (upper pictures) and an anti-ADPr antibody (lower pictures). Representative pictures for each condition are shown on the left; the quantification of nuclear (left) and mitochondrial (right) IF signals detected with the anti-ADPr antibody is depicted on the right.

(F) U2OS cells were transfected with siRNA targeting NMNAT1 or 2 and treated with H_2O_2, and nuclear ADP-ribosylation was analyzed via IF using an anti-PAR antibody (upper pictures) and an anti-ADPr antibody (lower pictures). Quantification of the signals detected by the anti-ADPr antibody (left) and the anti-PAR antibody (right) are shown in the lower panel. The quantifications of all signals (mitochondrial and nuclear) were normalized as described in Figure 1, and the y axes of all plots are depicted as log10 scale. Scale bars indicate 20 μm. For statistical analysis, a Student's t test was performed (n = 3–5; $^*p < 0.05$; $^{**}p < 0.005$; $^{***}p < 0.0005$).

CellPress
OPEN ACCESS

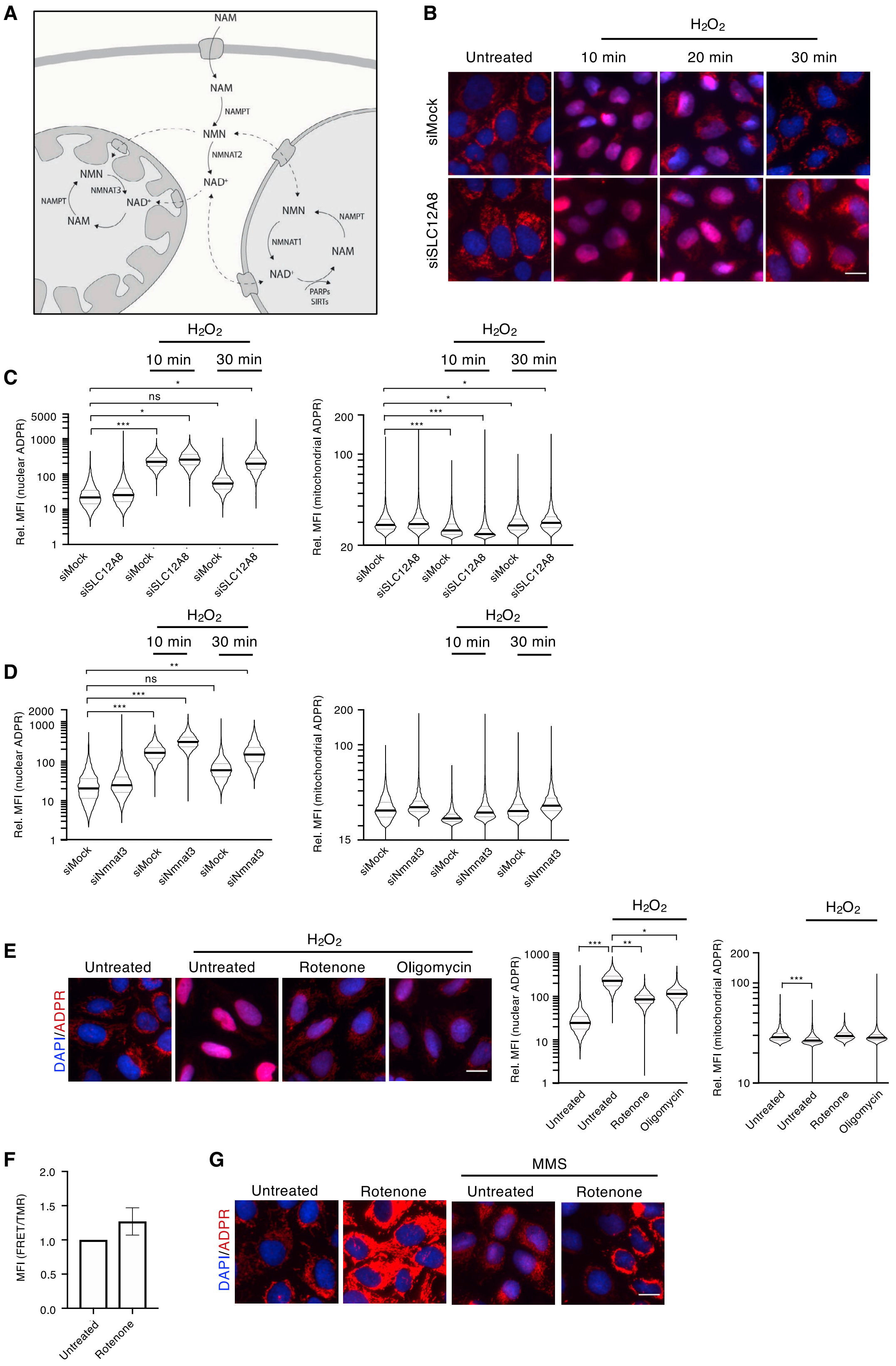

(legend on next page)

the cellular transport of NMN. Under these conditions, it is likely that a considerable fraction of NAM is converted to NMN, which is subsequently converted to NAD^+ by NMNAT1/2 and immediately (re-)consumed by ARTD1. Intriguingly, knockdown of SLC12A8 did not lead to long-term defects in the re-establishment of mitochondrial ADP-ribosylation following H_2O_2 treatment, suggesting that the mitochondrial NAD^+ levels are not dependent on the import of exogenous NMN (Figures 4B and 4C). To further investigate the restoration of mitochondrial ADP-ribosylation after H_2O_2 treatment, we individually knocked down NMNAT1, 2, or 3, treated cells with H_2O_2, and quantified nuclear and mitochondrial ADP-ribosylation. Knockdown of NMNAT3 but not NMNAT1 or 2 resulted in extended PARylation following H_2O_2 treatment, thus phenocopying the knockdown of SLC12A8 after 30 min (Figure 4D; Figure S6A). In addition, knockdown of NMNAT3 boosted H_2O_2-induced nuclear ADP-ribosylation relative to siMock samples even 10 min post-treatment, suggesting that inhibiting the mitochondrial NAD^+ salvage pathway might induce a general redistribution of subcellular NAD^+ pools. This result is in line with the data presented above (Figure 1D), where knockdown of NMNAT3 resulted in the redistribution of ADP-ribosylation signals from the mitochondria toward the cytoplasm and nucleus. Interestingly, knockdown of neither NMNAT1, nor NMNAT2, nor both had striking effects on either mitochondrial or nuclear ADP-ribosylation at any measured time point after H_2O_2 treatment. Similar to SLC12A8 knockdown, knockdown of NMNAT3 also had no effect on mitochondrial ADP-ribosylation dynamics. These findings strongly support the existence of a separate mitochondrial NAD^+ transporter (Davila et al., 2018; Girardi et al., 2020; Kory et al., 2020; Luongo et al., 2020) and suggest that both transporters (NMN and NAD^+) are important and necessary for the establishment and regulation of different subcellular NAD^+ pools.

Inhibition of the Respiratory Chain or F0F1 ATP Synthase Reduces H_2O_2- and MMS-Induced Nuclear ADP-Ribosylation

Based on the findings presented above, which suggest that H_2O_2 treatment reciprocally alters mitochondrial and nuclear ADP-ribosylation, we hypothesized that pre-treatment of cells with respiratory chain inhibitors that induce mitochondrial ADP-ribosylation would dampen H_2O_2-induced nuclear ADP-ribosylation. Indeed, pre-treatment of cells with rotenone, oligomycin, myxothiazol, or antimycin prior to H_2O_2 treatment significantly reduced nuclear ADP-ribosylation in U2OS cells, as well as in other tested cell lines (Figure 4E; Figures S6B–S6D, respectively). Interestingly, compared to H_2O_2 treatment only, pre-treatment with respiratory chain inhibitors led to a partial maintenance of mitochondrial ADP-ribosylation, even after H_2O_2 treatment (Figure 4E), which suggests that enhanced mitochondrial ADP-ribosylation after inhibitor treatment reduces the nuclear NAD^+ pool available for nuclear ADP-ribosylation. Based on this observation, we hypothesized that inhibition of respiration alters mitochondrial NAD^+ levels. To test the impact of respiratory chain inhibitors on mitochondrial NAD^+ levels, we used the same stable U2OS Flp-In T-Rex cells expressing the NAD^+ sensors described above (Figure S5D). These cells were treated with rotenone, and NAD^+ sensor FRET ratios were measured. Interestingly, rotenone treatment markedly increased mitochondrial NAD^+ levels (Figure 4F), suggesting that mitochondria are able to compensate for the lack of respiratory chain-mediated NAD^+ restoration by increasing mitochondrial NAD^+ levels, likely via direct uptake. Indeed, this could result in the observed increase in mitochondrial ADP-ribosylation and reduced mitochondrial NAD^+ release following H_2O_2 treatment that, subsequently, would dampen *de novo* nuclear ADP-ribosylation (Figure 4E). To investigate whether the effect respiratory chain inhibition had on nuclear ADP-ribosylation was specific to H_2O_2, we induced nuclear ADP-ribosylation with methyl methanesulfonate (MMS) alone or in combination with rotenone. Interestingly, similar to H_2O_2 treatment, rotenone pre-treatment also reduced MMS-induced nuclear ADP-ribosylation (Figure 4G).

Moreover, to investigate whether the increase in mitochondrial ADP-ribosylation would affect nuclear genotoxic stress responses beyond decreasing nuclear ADP-ribosylation, we pre-treated cells with rotenone and then stained for the DNA-damage marker γH2AX at various time points after H_2O_2 treatment (Figure S6E). Pre-treatment with rotenone completely abolished the increase in γH2AX signal following H_2O_2 treatment. As the phosphorylation reaction requires ATP as a substrate, the observed decrease in γH2AX levels suggests a general drop in cellular ATP following rotenone treatment (Li et al., 2003; Midzak et al., 2011). Interestingly, some subunits of the ATP synthase are among the most dominant mitochondrial ADP-ribosylation targets (Figure 2C), pointing toward an ADP-ribosylation-dependent regulation of the ATP synthase activities.

ADP-Ribosylation Inhibitors or Lack of ARTD1 Do Not Affect Mitochondrial ADP-Ribosylation

ADP-ribosylation gained considerable attention when PARP inhibitors were successfully used as anti-cancer treatments for

Figure 4. Lack of NMNAT3 and the NMN Transporter SLC12A8 Extends Nuclear ADP-Ribosylation after H_2O_2 Treatment

(A) Schematic overview of compartmentalized NAD^+ synthesis and breakdown in most transformed cells.

(B and C) U2OS cells were transfected with siRNA targeting SLC12A8, and nuclear (left) or mitochondrial (right) ADP-ribosylation was analyzed via IF and quantified at various time points following H_2O_2 treatment.

(D) NMNAT3 was knocked down in U2OS, and nuclear (left) or mitochondrial (right) ADP-ribosylation was analyzed via IF using an anti-ADPr antibody.

(E) U2OS cells were pre-treated for 1 h with rotenone or oligomycin prior to a 10-min treatment with H_2O_2 and nuclear and mitochondrial ADP-ribosylation was analyzed via IF.

(F) Mitochondrial NAD^+ levels following rotenone treatment were assessed in stable U2OS Flp-In T-Rex cells expressing an inducible mitochondrial NAD^+ sensor and analyzed via flow cytometry. Increased FRET ratios correspond to increased NAD^+ levels. FRET ratios of three independent experiments including square deviation are shown.

(G) IF using the anti-ADPr antibody of U2OS cells pre-treated with rotenone and subsequently treated for 3 h with MMS. The quantifications of all signals (mitochondrial and nuclear) were normalized as described in Figure 1, and the y axes of all plots are depicted as log10 scale. Scale bars indicate 20 μm. For statistical analysis, a Student's t test was performed ($n = 3–5$; *$p < 0.05$; **$p < 0.005$; ***$p < 0.0005$).

CellPress
OPEN ACCESS

patients harboring BRCA-deficient tumors (Farmer et al., 2005). To test whether mitochondrial ADP-ribosylation levels influence the efficacy of PARP inhibitors, U2OS cells were treated with different PARP inhibitors (Figure 5A) at concentrations known to inhibit nuclear PARylation (Figure S7A). Quantification of the mitochondrial ADP-ribosylation signals revealed that mitochondrial ADPr modifications do not appear to be affected by PARP inhibitor treatment (Figure 5A). Despite previous reports demonstrating that a minor fraction of ARTD1 localizes to mitochondria (Rossi et al., 2009; Szczesny et al., 2014), our findings suggest that neither ARTD1 nor ARTD2 (PARP2) were directly responsible for the mitochondrial ADP-ribosylation observed here. To validate these findings, we repeated the IF analysis with the anti-ADP-ribosylation antibody on ARTD1 knockout ($^{-/-}$) U2OS cells (Hanzlikova et al., 2017) (Figure 5B). When compared to rotenone treatment alone, we found that mitochondrial ADP-ribosylation increased slightly in U2OS cells following ARTD1 depletion, confirming that ARTD1 most likely does not contribute to the mitochondrial ADP-ribosylation. Moreover, the increased mitochondrial ADP-ribosylation levels observed upon ARTD1 depletion are in line with previous reports, which suggested that a fraction of cellular NAD^+ is consumed by ARTD1 under basal conditions; thus ARTD1 depletion increases the amount of NAD^+ available for other NAD^+-consuming enzymes within the cell (Liu et al., 2018).

NAD^+ Compartmentalization Limits ARTD1 Chromatin Retention and PARP Inhibitor Efficacy

While ARTD1 activity modulates intracellular NAD^+ availabilities, NAD^+ compartmentalization and availability also regulate ARTD1 (Alano et al., 2004; Módis et al., 2012). Previous studies have shown that H_2O_2-induced nuclear ADP-ribosylation regulates ARTD1-chromatin interaction (Michelena et al., 2018; Wacker et al., 2007). This led us to question whether respiratory chain inhibition and the resulting re-distribution of intracellular NAD^+ toward mitochondria might enhance ARTD1 chromatin retention. U2OS cells were thus treated with MMS alone or co-treated with either olaparib or rotenone for 2 h, and chromatin-bound ARTD1 levels were assessed via IF on pre-extracted cells (Figure 5C). While individual MMS, olaparib, or rotenone treatments did not alter chromatin-bound ARTD1 in a detectable manner, co-treatment of cells with MMS and rotenone increased the amount of chromatin-bound ARTD1 similar to co-treatment of cells with MMS and olaparib (i.e., control). These results indicate that NAD^+ compartmentalization and intracellular availability indeed modulate nuclear ARTD1 chromatin retention. The same trend was also observed with another respiratory chain inhibitor, antimycin (Figure S7B).

PARP inhibitors have been reported to inhibit nuclear ADP-ribosylation after H_2O_2 treatment (Andersson et al., 2016; Michelena et al., 2018) (Figure 5D). As their inhibitory capacities are believed to depend on intracellular NAD^+ levels, we hypothesized that NAD^+ release from mitochondria could also dampen PARP inhibitor efficacy. U2OS cells were thus treated with carbonyl cyanide-*4*-(trifluoromethoxy) phenylhydrazone (FCCP), a mitochondrial protonophore that uncouples mitochondrial inner membrane potential, reduces the pH in the matrix, and induces mitochondrial ROS formation (Abad et al., 2004; Hung et al., 2018). FCCP treatment indeed reduced mitochondrial NAD^+ levels in a time-dependent manner to a similar extent as H_2O_2 (Figure S7C). Concomitantly, FCCP treatment weakly reduced mitochondrial ADP-ribosylation signals (Figure S7D), and pre-treatment of cells with FCCP dampened PARP inhibitor efficacy (Figure 5D). Moreover, inhibitor concentrations had to be increased 5-fold to inhibit H_2O_2-induced nuclear PARylation compared to the concentrations required to inhibit nuclear ADP-ribosylation in the absence of FCCP (Figure 5D). Importantly, this phenomenon was observed for three different PARP inhibitors: PJ34, olaparib, and veliparib (Figure 5D; Figure S7E). Taken together, these findings suggest that mitochondrial permeability and the resulting changes in cytoplasmic and nuclear NAD^+ concentrations alter ARTD1 chromatin retention and PARP inhibitor efficacy.

DISCUSSION

Here we aimed to define and characterize mitochondrial ADP-ribosylation, elucidate how it is regulated, and determine how this would influence the ADP-ribosylation potential in other subcellular compartments. We demonstrated that mitochondrial ADP-ribosylation is indeed present and detectable using different antibodies or an engineered Af1521 macrodomain-FC fusion protein and via several methods (IF, WB, MS).

We further found that mitochondrial ADP-ribosylation is strongly dependent on NAD^+ availability, responds to metabolic stimulation, and is reversible and dynamic. Interestingly, we provided further evidence that strong mitochondrial and nuclear ADP-ribosylation occur in an almost mutually exclusive manner, which is likely mediated by NAD^+ shuttling. Indeed, upon strong nuclear NAD^+ demand, mitochondria appear to play an important role by providing the NAD^+ required for nuclear PARylation. Finally, our data demonstrate that NAD^+ release from the mitochondria reduces PARP inhibitor efficacy, likely via competition, indicating that PARP inhibitor efficacy and their therapeutic potentials may be tightly linked to subcellular NAD^+ distributions.

Using two different anti-ADPr antibodies, we were able to detect basal mitochondrial ADP-ribosylation via IF and WB. Although the antibodies also recognize PARylated proteins, the absence of any mitochondrial staining when using an anti-PAR antibody suggests that the detected mitochondrial ADP-ribosylation signals rather represent ADPr monomers or short ADPr oligomers. This is further strengthened by the fact that PARG inhibition had no effect on mitochondrial ADP-ribosylation (Figure S7F), which is in line with a previous study (Niere et al., 2012). Furthermore, cycloheximide treatment confirmed that the signal observed was dynamic and protein-synthesis dependent. To exclude that the signals detected here were the result of cross-reactivity, we tested the specificity of our antibody by dot blot with several different adenosine derivates. Moreover, serine, threonine, and tyrosine AMPylated peptides/proteins were not identified in the ADP-ribosylome MS data presented here, and knockdown of SelO that catalyzes mitochondrial AMPylation did not affect mitochondrial ADP-ribosylation. Finally, NAMPT knockdown experiments provided further evidence that the observed mitochondrial signal was highly dependent on intracellular NAD^+ levels.

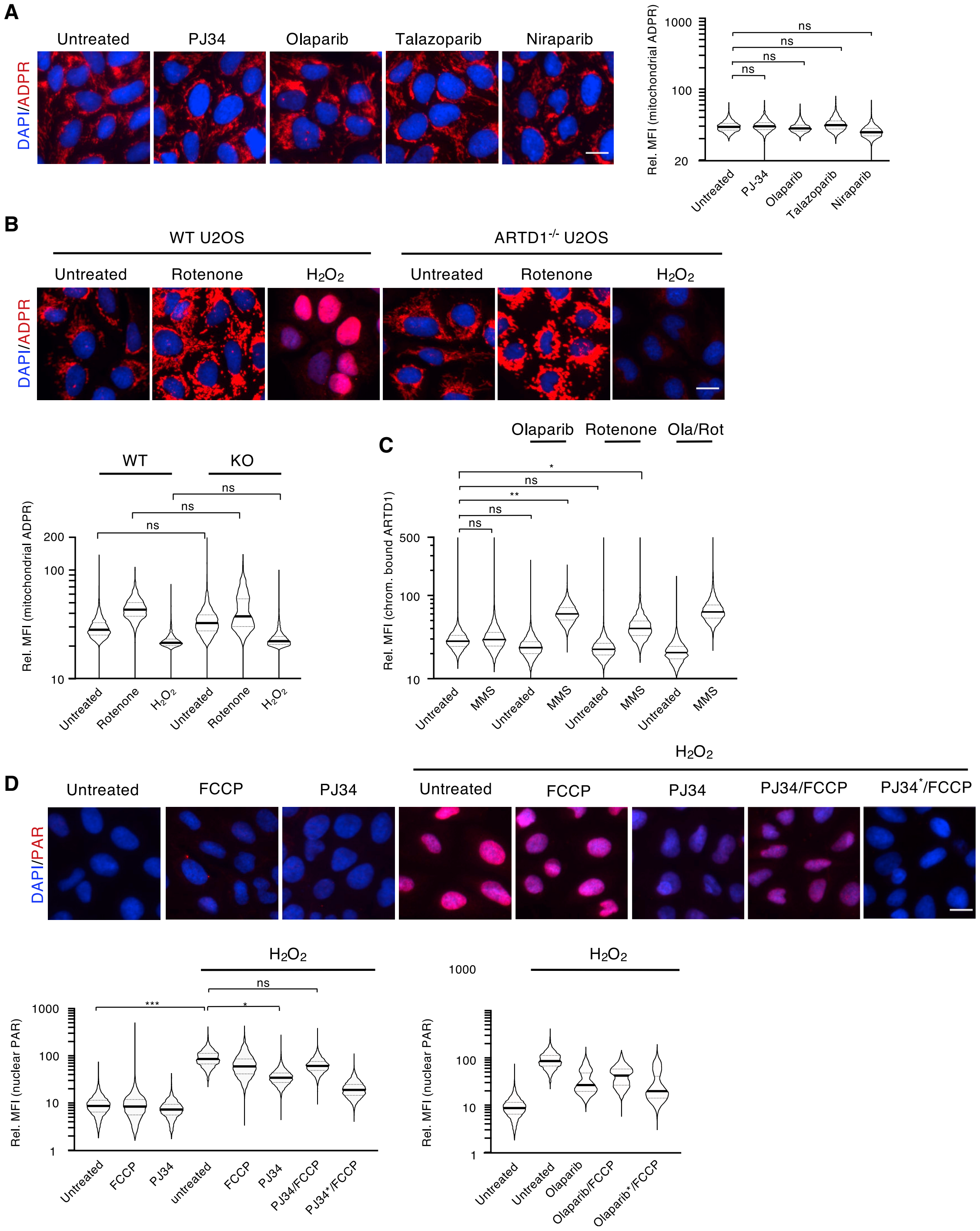

Figure 5. Release of Mitochondrial NAD+ Antagonizes PARP Inhibitor Treatment

(A) U2OS cells were treated with PJ34, olaparib, talazoparib, or niraparib, and mitochondrial ADP-ribosylation was subsequently assessed via IF using the anti-ADPr antibody.

(B) U2OS ARTD1$^{-/-}$ cells were treated with either rotenone or H_2O_2, and the mitochondrial and nuclear ADP-ribosylation were assessed via IF with our anti-ADPr antibody.

(C) U2OS cells were co-treated with MMS and olaparib, rotenone or a combination for 2 h and after pre-extraction, chromatin-bound ARTD1 was analyzed via IF.

(D) U2OS cells were pre-treated with PJ34 or olaparib, then subjected to an FCCP pulse followed by H_2O_2 and analyzed via IF using an anti-PAR antibody. * = 5-fold increased concentration of inhibitors. Representative pictures are shown in the upper panel; the quantification of the nuclear PAR signal is shown in the lower panel. The quantifications of all signals (mitochondrial and nuclear) were normalized as described in Figure 1, and the y axes of all plots are depicted as log10 scale. Scale bars indicate 20 μm.

The fact that the observed mitochondrial signal is present under basal conditions in all cell types investigated here suggests that these modifications may be functionally beneficial for the cell. While we show that NMN supplementation increased mitochondrial ADP-ribosylation, it would be interesting to correlate our findings with the increase in cell viability (Grozio et al., 2019) and mitigation of age-associated decline (Mills et al., 2016) that have been recently observed upon NMN supplementation. The changes in mitochondrial ADP-ribosylation observed following metabolic stress (e.g., respiratory chain inhibition) further indicate that this modification might regulate mitochondrial functions in a metabolic condition-dependent manner. This assumption is supported by our MS-based data, which identified several ADP-ribosylated proteins and protein complexes that are involved in key metabolic processes. Although direct proof was so far missing, many of the modified proteins (e.g., subunits of respiratory chain complex I or the ATP synthase) were proposed to be associated with ADP-ribose in a previous study (Gagné et al., 2008). As inhibition of both these complexes increases mitochondrial ADP-ribosylation, it is intriguing to speculate that modifying these complexes might directly modulate their activity.

Contrary to the strong mitochondrial ADP-ribosylation signal observed via IF in U2OS cells, our MS-based ADP-ribosylome analysis of these cells revealed that the majority of ADP-ribosylated proteins identified here are abundant nuclear and/or cytoplasmic proteins. Indeed, we only identified six ADP-ribosylated mitochondrial proteins in these cells. We believe that this is due to the fact that cultured cells typically have low mitochondrial loads, which also means that these proteins are very low in abundance in the cell relative to cytoplasmic/nuclear proteins. This is supported by a recent study that applied an alternative MS-based methodology to define ADP-ribosylated peptides/proteins (Buch-Larsen et al., 2020). Here, the mitochondrial ADP-ribosylome represented only 10% of the total physiological/baseline ADP-ribosylome identified in HeLa cells, and all of these mitochondrial proteins were low in abundance. Thus, closing this general protein dynamic range gap by shifting our ADP-ribosylome MS analysis workflow from cells to tissues with high mitochondrial loads (skeletal muscle) allowed us to identify significantly more ADP-ribosylated mitochondrial proteins. The identified mitochondrial ADP-ribosylome comprised > 25% of the total ADP-ribosylome. This stark increase in the number of mitochondrial ADP-ribosylated proteins, as well as ADP-ribosylated proteins that localize to other organelles (ER, Golgi, cytoskeleton), identified relative to cytoplasmic/nuclear ADP-ribosylated proteins in tissues (56%) compared to cell culture cells (23%), clearly indicate that narrowing the overall cytoplasmic/nuclear protein abundance dynamic range improved the identification rates of low-abundant ADP-ribosylated proteins.

Interestingly, ADP-ribosylation in different subcellular compartments (here mainly nucleus and mitochondria) seems to be highly interconnected. The H_2O_2-induced reduction in mitochondrial ADP-ribosylation preceded and correlated with the appearance of nuclear ADP-ribosylation, demonstrating for the first time an inter-compartmental ADP-ribosylation cross-talk between the nucleus and mitochondria. Given that the majority of the mitochondrial ADP-ribosylated proteins reside within either the matrix or the inner membrane, it is rather unlikely that the mitochondrial signal loss observed after H_2O_2 treatment could be explained by retrograde protein shuttling. In line with this assumption, none of the mitochondrial ADP-ribosylated proteins identified here are currently known to translocate to the nucleus upon genotoxic stress, neither prior to nor following H_2O_2 treatment (Martello et al., 2016). Based on the compartmentalized NAD^+ measurements we performed, the change in signal compartmentalization might rather be explained by a redistribution of NAD^+ and a very fast reversible protein ADP-ribosylation. In fact, a recent publication already reported that a slight redistribution of NAD^+ between cytoplasm and nucleus could modulate gene expression by regulating ARTD1-mediated ADP-ribosylation (Ryu et al., 2018). Since H_2O_2 induces ARTD1 hyper-activation, it seems coherent that nuclear NAD^+ levels might not be sufficient to fuel the drastic amount of ADP-ribosylation generated under these conditions. Indeed, a recent study monitoring NAD^+ fluxes in various cells and organs demonstrated that upon induction of DNA damage, cells experience a considerable ARTD1-dependent loss of NAD^+, accounting for around a third of the total NAD^+ (Liu et al., 2018). As nuclear NAD^+ levels are described to be around 7–8 times lower compared to those observed in mitochondria (Williamson et al., 1967; Zhang et al., 2002), it is very unlikely that the drop in cellular NAD^+ is derived only from the nuclear NAD^+ metabolism. Accordingly, knockdown of neither NMNAT1, NMNAT2, or both resulted in a considerable decrease of H_2O_2-induced nuclear ADP-ribosylation. We therefore propose that, under the conditions tested here, mitochondrial NAD^+ is released to sustain proper nuclear ADP-ribosylation in response to the encountered stress. Due to hyper-activation of ARTD1, a high concentration of NAM is produced, which could also be rapidly converted to NMN by NMNAT1/2.

In support of this hypothesis, we found that enhanced mitochondrial ADP-ribosylation prior to H_2O_2 treatment dampened the nuclear ADP-ribosylation. The rapid change in ADP-ribosylation between the mitochondria and the nucleus, as well as the rapid decrease in mitochondrial NAD^+ 1 min after H_2O_2 treatment, suggest an active release of NAD^+ from the mitochondria. Our results indicate that restoration of mitochondrial NAD^+ can be mediated by NMN uptake. Moreover, recent studies identified MCART1/SLC25A51 as the mitochondrial NAD^+ transporter responsible for direct uptake of NAD^+ (Davila et al., 2018; Girardi et al., 2020; Kory et al., 2020). Together, these findings strongly indicate that mitochondria can regulate their NAD^+ levels via different, partially redundant mechanisms.

Finally, we observe that mitochondrial and nuclear NAD^+ availability modulates ARTD1 chromatin retention, as well as PARP inhibitor efficacy. Thus, mitochondrial fitness and mitochondrial NAD^+ levels might need to be considered when deciding to treat tumors with this class of compounds. In agreement with this conclusion, the combination of a NAMPT small-molecule inhibitor, FK866, with olaparib inhibited triple-negative (TN) breast tumor growth *in vivo* to a greater extent than either single agent alone, suggesting that assessing NAMPT/PARP inhibitor combinations for the treatment of TN breast cancer may be warranted (Bajrami et al., 2012). Cancer cells very often induce the Warburg

effect (Burns and Manda, 2017), which would decrease the requirement for NAD^+ in the mitochondria and provide a valuable NAD^+ source for other NAD^+-dependent processes such as the DNA damage response in the nucleus (Gomes et al., 2013). Our results may, therefore, have medical implications, as they suggest that mitochondrial fitness and integrity should be considered when treating cancer patients.

As mitochondrial ADP-ribosylation might influence NAD^+-dependent processes in other subcellular compartments, it is crucial to identify the writers and erasers involved in mitochondrial ADP-ribosylation turnover. The KD of a potential mitochondrial writer was reported to be lower compared to nuclear ARTD1 (KD around 20 μM compared to 70 μM) (Brunyanszki et al., 2016), suggesting that even under conditions that reduce the NAD^+/NADH ratio within the mitochondria (e.g., inhibition of respiration), ADP-ribosylation can occur. Although ARTD1 has been shown to co-purify with mitochondria, our data exclude a contribution of ARTD1 to mitochondrial ADP-ribosylation in U2OS cells. This is in agreement with the finding that the currently available PARP inhibitors were not able to inhibit mitochondrial ADP-ribosylation. SIRT4 is another enzyme previously described to specifically ADP-ribosylate the glutamate dehydrogenase (GDH) and thereby regulate its activity (Ahuja et al., 2007; Haigis et al., 2006). However, since GDH is so far the only identified ADP-ribosylated target of SIRT4 among the many modified mitochondrial proteins, it is unlikely that SIRT4 is the main responsible enzyme for the observed mitochondrial ADP-ribosylation. In line with this, knockdown of SIRT4 in U2OS cells did not reduce mitochondrial ADP-ribosylation analyzed by IF. Moreover, since we identified different ADPr-acceptor sites that differ in their intra-mitochondrial localization (matrix, inner-, or outer membrane), we would hypothesize that more than one enzyme might be responsible for catalyzing mitochondrial ADP-ribosylation.

STAR★METHODS

Detailed methods are provided in the online version of this paper and include the following:

- KEY RESOURCES TABLE
- RESOURCE AVAILABILITY
  - Lead Contact
  - Materials availability
  - Data and code availability
- EXPERIMENTAL MODEL AND SUBJECT DETAILS
  - Cell lines
- METHOD DETAILS
  - Cell culture
  - Drug treatments
  - siRNA transfection
  - Immunostaining
  - Confocal microscopy
  - ELISA
  - *In vitro* ADP-ribosylation assay
  - Rapid isolation of mitochondria from cells
  - Western- and Dot blotting
  - ADPr-Peptide Enrichment
- QUANTIFICATION AND STATISTICAL ANALYSIS
  - Quantitative image-based cytometry (QIBC)
  - Statistical Analysis
  - Liquid Chromatography and Mass Spectrometry Analysis
  - Raw Mass Spectrometry Data Analysis
  - Intracellular, compartmentalized NAD^+ measurements

SUPPLEMENTAL INFORMATION

Supplemental Information can be found online at https://doi.org/10.1016/j.molcel.2020.12.034.

ACKNOWLEDGMENTS

We thank Tobias Suter (University of Zurich) for helpful discussions and for providing editorial assistance. We thank Massimiliano Manzo (University of Zurich) for suggestions on the statistical analysis. We thank Birgit Koch (Max Planck Institute for Medical Research) for developing and sorting the stable U2OS Flp-In T-Rex cell lines. ARTD1/PARP1 knockout U2OS cells were kindly provided by Keith Caldecott (Genome Damage and Stability Centre, University of Sussex, UK) and the anti-pan-ADPr Ab by Cell Signaling Technology (Massachusetts, USA). We thank the Center for Microscopy and Image Analysis and the Functional Genomics Center of the University of Zurich for services and assistance. F.T. and M.A. were funded by the Swiss National Science Foundation (Grant PP00P3_179057 to M.A.) and the European Research Council (ERC) under the European Union's Horizon 2020 research and innovation program (Grant 714326 to M.A.). ADP-ribosylation research in the laboratory of M.O.H. is funded by the Kanton of Zurich and the Swiss National Science Foundation (grants 310030_157019 and 31003A_176177).

AUTHOR CONTRIBUTIONS

Project conceptualization and administration: M.O.H. and A.-K.H. (lead) and M.A. (supporting). Data curation and formal analysis: A.-K.H. (lead) and F.T. (supporting); D.M.L.P. (lead MS part). Investigation (specific experiments): A.-K.H. (lead), F.T., C.G., F.R., L.B., K.N., A.H., P.G.A.P., L.M. (supporting); D.M.L.P. (lead MS part) and P.G.A.P. (supporting). Visualization and validation: A.-K.H. (lead), D.M.L.P. (supporting). Methodology: A.-K.H. (lead) and M.A., F.T., C.G., F.R., K.J., P.G.A.P. (supporting); D.M.L.P. (lead MS part); P.G.A.P. (lead bioinformatics) and D.M.L.P. (supporting). Writing, review & editing: M.O.H., A.-K.H., D.M.L.P. (lead), M.A. (supporting), F.T., C.G., F.R., L.B., K.J., and P.G.A.P. (editing).

DECLARATION OF INTERESTS

The authors declare no financial interest.

Received: April 7, 2020
Revised: October 30, 2020
Accepted: December 18, 2020
Published: January 14, 2021

REFERENCES

Abad, M.F., Di Benedetto, G., Magalhães, P.J., Filippin, L., and Pozzan, T. (2004). Mitochondrial pH monitored by a new engineered green fluorescent protein mutant. J. Biol. Chem. *279*, 11521–11529.

Abplanalp, J., Leutert, M., Frugier, E., Nowak, K., Feurer, R., Kato, J., Kistemaker, H.V.A., Filippov, D.V., Moss, J., Caflisch, A., and Hottiger, M.O. (2017). Proteomic analyses identify ARH3 as a serine mono-ADP-ribosylhydrolase. Nat. Commun. *8*, 2055.

Ahuja, N., Schwer, B., Carobbio, S., Waltregny, D., North, B.J., Castronovo, V., Maechler, P., and Verdin, E. (2007). Regulation of insulin secretion by SIRT4, a mitochondrial ADP-ribosyltransferase. J. Biol. Chem. *282*, 33583–33592.

CellPress
OPEN ACCESS

Alano, C.C., Ying, W., and Swanson, R.A. (2004). Poly(ADP-ribose) polymerase-1-mediated cell death in astrocytes requires NAD+ depletion and mitochondrial permeability transition. J. Biol. Chem. *279*, 18895–18902.

Alano, C.C., Tran, A., Tao, R., Ying, W., Karliner, J.S., and Swanson, R.A. (2007). Differences among cell types in NAD(+) compartmentalization: a comparison of neurons, astrocytes, and cardiac myocytes. J. Neurosci. Res. *85*, 3378–3385.

Altmeyer, M., Toledo, L., Gudjonsson, T., Grøfte, M., Rask, M.B., Lukas, C., Akimov, V., Blagoev, B., Bartek, J., and Lukas, J. (2013). The chromatin scaffold protein SAFB1 renders chromatin permissive for DNA damage signaling. Mol. Cell *52*, 206–220.

Andersson, A., Bluwstein, A., Kumar, N., Teloni, F., Traenkle, J., Baudis, M., Altmeyer, M., and Hottiger, M.O. (2016). PKCα and HMGB1 antagonistically control hydrogen peroxide-induced poly-ADP-ribose formation. Nucleic Acids Res. *44*, 7630–7645.

Bajrami, I., Kigozi, A., Van Weverwijk, A., Brough, R., Frankum, J., Lord, C.J., and Ashworth, A. (2012). Synthetic lethality of PARP and NAMPT inhibition in triple-negative breast cancer cells. EMBO Mol. Med. *4*, 1087–1096.

Bilan, V., Leutert, M., Nanni, P., Panse, C., and Hottiger, M.O. (2017). Combining Higher-Energy Collision Dissociation and Electron-Transfer/Higher-Energy Collision Dissociation Fragmentation in a Product-Dependent Manner Confidently Assigns Proteomewide ADP-Ribose Acceptor Sites. Anal. Chem. *89*, 1523–1530.

Bogan, K.L., and Brenner, C. (2008). Nicotinic acid, nicotinamide, and nicotinamide riboside: a molecular evaluation of NAD+ precursor vitamins in human nutrition. Annu. Rev. Nutr. *28*, 115–130.

Brunyanszki, A., Szczesny, B., Virág, L., and Szabo, C. (2016). Mitochondrial poly(ADP-ribose) polymerase: The Wizard of Oz at work. Free Radic. Biol. Med. *100*, 257–270.

Buch-Larsen, S.C., Hendriks, I.A., Lodge, J.M., Rykær, M., Furtwängler, B., Shishkova, E., Westphall, M.S., Coon, J.J., and Nielsen, M.L. (2020). Mapping Physiological ADP-Ribosylation Using Activated Ion Electron Transfer Dissociation. Cell Rep. *32*, 108176.

Burns, J.S., and Manda, G. (2017). Metabolic Pathways of the Warburg Effect in Health and Disease: Perspectives of Choice, Chain or Chance. Int. J. Mol. Sci. *18*, 2755.

Calvo, S.E., Clauser, K.R., and Mootha, V.K. (2016). MitoCarta2.0: an updated inventory of mammalian mitochondrial proteins. Nucleic Acids Res. *44* (D1), D1251–D1257.

Cambronne, X.A., Stewart, M.L., Kim, D., Jones-Brunette, A.M., Morgan, R.K., Farrens, D.L., Cohen, M.S., and Goodman, R.H. (2016). Biosensor reveals multiple sources for mitochondrial NAD^+. Science *352*, 1474–1477.

Chen, W.W., Freinkman, E., Wang, T., Birsoy, K., and Sabatini, D.M. (2016). Absolute Quantification of Matrix Metabolites Reveals the Dynamics of Mitochondrial Metabolism. Cell *166*, 1324–1337.e1311.

Chiarugi, A., Dölle, C., Felici, R., and Ziegler, M. (2012). The NAD metabolome–a key determinant of cancer cell biology. Nat. Rev. Cancer *12*, 741–752.

Davila, A., Liu, L., Chellappa, K., Redpath, P., Nakamaru-Ogiso, E., Paolella, L.M., Zhang, Z., Migaud, M.E., Rabinowitz, J.D., and Baur, J.A. (2018). Nicotinamide adenine dinucleotide is transported into mammalian mitochondria. eLife *7*, 7.

Di Lisa, F., Menabò, R., Canton, M., Barile, M., and Bernardi, P. (2001). Opening of the mitochondrial permeability transition pore causes depletion of mitochondrial and cytosolic NAD+ and is a causative event in the death of myocytes in postischemic reperfusion of the heart. J. Biol. Chem. *276*, 2571–2575.

Farmer, H., McCabe, N., Lord, C.J., Tutt, A.N., Johnson, D.A., Richardson, T.B., Santarosa, M., Dillon, K.J., Hickson, I., Knights, C., et al. (2005). Targeting the DNA repair defect in BRCA mutant cells as a therapeutic strategy. Nature *434*, 917–921.

Gagné, J.-P., Isabelle, M., Lo, K.S., Bourassa, S., Hendzel, M.J., Dawson, V.L., Dawson, T.M., and Poirier, G.G. (2008). Proteome-wide identification of poly(ADP-ribose) binding proteins and poly(ADP-ribose)-associated protein complexes. Nucleic Acids Res. *36*, 6959–6976.

Girardi, E., Fiume, G., Goldmann, U., Sin, C., Müller, F., Lindinger, S., Sedlyrov, V., Srndic, I., Agerer, B., Kartnig, F., et al. (2020). A systematic genetic interaction map of human solute carriers assigns a role to SLC25A51/MCART1 in mitochondrial NAD uptake. bioRxiv.

Gomes, A.P., Price, N.L., Ling, A.J., Moslehi, J.J., Montgomery, M.K., Rajman, L., White, J.P., Teodoro, J.S., Wrann, C.D., Hubbard, B.P., et al. (2013). Declining NAD(+) induces a pseudohypoxic state disrupting nuclear-mitochondrial communication during aging. Cell *155*, 1624–1638.

Grozio, A., Mills, K.F., Yoshino, J., Bruzzone, S., Sociali, G., Tokizane, K., Lei, H.C., Cunningham, R., Sasaki, Y., Migaud, M.E., and Imai, S.I. (2019). Slc12a8 is a nicotinamide mononucleotide transporter. Nat Metab *1*, 47–57.

Ha, H.C., and Snyder, S.H. (1999). Poly(ADP-ribose) polymerase is a mediator of necrotic cell death by ATP depletion. Proc. Natl. Acad. Sci. USA *96*, 13978–13982.

Haigis, M.C., and Sinclair, D.A. (2010). Mammalian sirtuins: biological insights and disease relevance. Annu. Rev. Pathol. *5*, 253–295.

Haigis, M.C., Mostoslavsky, R., Haigis, K.M., Fahie, K., Christodoulou, D.C., Murphy, A.J., Valenzuela, D.M., Yancopoulos, G.D., Karow, M., Blander, G., et al. (2006). SIRT4 inhibits glutamate dehydrogenase and opposes the effects of calorie restriction in pancreatic beta cells. Cell *126*, 941–954.

Hanzlikova, H., Gittens, W., Krejcikova, K., Zeng, Z., and Caldecott, K.W. (2017). Overlapping roles for PARP1 and PARP2 in the recruitment of endogenous XRCC1 and PNKP into oxidized chromatin. Nucleic Acids Res. *45*, 2546–2557.

Horenstein, A.L., Sizzano, F., Lusso, R., Besso, F.G., Ferrero, E., Deaglio, S., Corno, F., and Malavasi, F. (2009). CD38 and CD157 ectoenzymes mark cell subsets in the human corneal limbus. Mol. Med. *15*, 76–84.

Hottiger, M.O. (2015). Nuclear ADP-Ribosylation and Its Role in Chromatin Plasticity, Cell Differentiation, and Epigenetics. Annu. Rev. Biochem. *84*, 227–263.

Hung, C.H., Cheng, S.S., Cheung, Y.T., Wuwongse, S., Zhang, N.Q., Ho, Y.S., Lee, S.M., and Chang, R.C. (2018). A reciprocal relationship between reactive oxygen species and mitochondrial dynamics in neurodegeneration. Redox Biol. *14*, 7–19.

Kanai, Y. (2016). Overview on poly(ADP-ribose) immuno-biomedicine and future prospects. Proc. Jpn. Acad., Ser. B, Phys. Biol. Sci. *92*, 222–236.

Katsyuba, E., and Auwerx, J. (2017). Modulating NAD^+ metabolism, from bench to bedside. EMBO J. *36*, 2670–2683.

Koch-Nolte, F., Fischer, S., Haag, F., and Ziegler, M. (2011). Compartmentation of NAD+-dependent signalling. FEBS Lett. *585*, 1651–1656.

Kory, N., Uit de Bos, J., van der Rijt, S., Jankovic, N., Güra, M., Arp, N., Pena, I.A., Prakash, G., Chan, S.H., Kunchok, T., et al. (2020). MCART1/SLC25A51 is required for mitochondrial NAD transport. Sci. Adv. *6*, eabe5310.

Kraus, W.L. (2015). PARPs and ADP-Ribosylation: 50 Years … and Counting. Mol. Cell *58*, 902–910.

Larsen, S.C., Hendriks, I.A., Lyon, D., Jensen, L.J., and Nielsen, M.L. (2018). Systems-wide Analysis of Serine ADP-Ribosylation Reveals Widespread Occurrence and Site-Specific Overlap with Phosphorylation. Cell Rep. *24*, 2493–2505.e2494.

Leutert, M., Menzel, S., Braren, R., Rissiek, B., Hopp, A.K., Nowak, K., Bisceglie, L., Gehrig, P., Li, H., Zolkiewska, A., et al. (2018). Proteomic Characterization of the Heart and Skeletal Muscle Reveals Widespread Arginine ADP-Ribosylation by the ARTC1 Ectoenzyme. Cell Rep. *24*, 1916–1929.e1915.

Li, N., Ragheb, K., Lawler, G., Sturgis, J., Rajwa, B., Melendez, J.A., and Robinson, J.P. (2003). Mitochondrial complex I inhibitor rotenone induces apoptosis through enhancing mitochondrial reactive oxygen species production. J. Biol. Chem. *278*, 8516–8525.

Li, B.B., Wang, X., Tai, L., Ma, T.T., Shalmani, A., Liu, W.T., Li, W.Q., and Chen, K.M. (2018). NAD Kinases: Metabolic Targets Controlling Redox Co-enzymes

and Reducing Power Partitioning in Plant Stress and Development. Front. Plant Sci. *9*, 379.

Liu, L., Su, X., Quinn, W.J., 3rd, Hui, S., Krukenberg, K., Frederick, D.W., Redpath, P., Zhan, L., Chellappa, K., White, E., et al. (2018). Quantitative Analysis of NAD Synthesis-Breakdown Fluxes. Cell Metab. *27*, 1067–1080.e1065.

Lukinavičius, G., Umezawa, K., Olivier, N., Honigmann, A., Yang, G., Plass, T., Mueller, V., Reymond, L., Corrêa, I.R., Jr., Luo, Z.G., et al. (2013). A near-infrared fluorophore for live-cell super-resolution microscopy of cellular proteins. Nat. Chem. *5*, 132–139.

Luongo, T.S., Eller, J.M., Lu, M.J., Niere, M., Raith, F., Perry, C., Bornstein, M.R., Oliphint, P., Wang, L., McReynolds, M.R., et al. (2020). SLC25A51 is a mammalian mitochondrial NAD^+ transporter. Nature *588*, 174–179.

Lüscher, B., Bütepage, M., Eckei, L., Krieg, S., Verheugd, P., and Shilton, B.H. (2018). ADP-Ribosylation, a Multifaceted Posttranslational Modification Involved in the Control of Cell Physiology in Health and Disease. Chem. Rev. *118*, 1092–1136.

Martello, R., Leutert, M., Jungmichel, S., Bilan, V., Larsen, S.C., Young, C., Hottiger, M.O., and Nielsen, M.L. (2016). Proteome-wide identification of the endogenous ADP-ribosylome of mammalian cells and tissue. Nat. Commun. *7*, 12917.

Meyer, T., and Hilz, H. (1986). Production of anti-(ADP-ribose) antibodies with the aid of a dinucleotide-pyrophosphatase-resistant hapten and their application for the detection of mono(ADP-ribosyl)ated polypeptides. Eur. J. Biochem. *155*, 157–165.

Michelena, J., Lezaja, A., Teloni, F., Schmid, T., Imhof, R., and Altmeyer, M. (2018). Analysis of PARP inhibitor toxicity by multidimensional fluorescence microscopy reveals mechanisms of sensitivity and resistance. Nat. Commun. *9*, 2678.

Midzak, A.S., Chen, H., Aon, M.A., Papadopoulos, V., and Zirkin, B.R. (2011). ATP synthesis, mitochondrial function, and steroid biosynthesis in rodent primary and tumor Leydig cells. Biol. Reprod. *84*, 976–985.

Mills, K.F., Yoshida, S., Stein, L.R., Grozio, A., Kubota, S., Sasaki, Y., Redpath, P., Migaud, M.E., Apte, R.S., Uchida, K., et al. (2016). Long-Term Administration of Nicotinamide Mononucleotide Mitigates Age-Associated Physiological Decline in Mice. Cell Metab. *24*, 795–806.

Módis, K., Gero, D., Erdélyi, K., Szoleczky, P., DeWitt, D., and Szabo, C. (2012). Cellular bioenergetics is regulated by PARP1 under resting conditions and during oxidative stress. Biochem. Pharmacol. *83*, 633–643.

Niere, M., Mashimo, M., Agledal, L., Dölle, C., Kasamatsu, A., Kato, J., Moss, J., and Ziegler, M. (2012). ADP-ribosylhydrolase 3 (ARH3), not poly(ADP-ribose) glycohydrolase (PARG) isoforms, is responsible for degradation of mitochondrial matrix-associated poly(ADP-ribose). J. Biol. Chem. *287*, 16088–16102.

Nowak, K., Rosenthal, F., Karlberg, T., Bütepage, M., Thorsell, A.G., Dreier, B., Grossmann, J., Sobek, J., Imhof, R., Lüscher, B., et al. (2020). Engineering Af1521 improves ADP-ribose binding and identification of ADP-ribosylated proteins. Nat. Commun. *11*, 5199.

Revollo, J.R., Grimm, A.A., and Imai, S. (2004). The NAD biosynthesis pathway mediated by nicotinamide phosphoribosyltransferase regulates Sir2 activity in mammalian cells. J. Biol. Chem. *279*, 50754–50763.

Rossi, M.N., Carbone, M., Mostocotto, C., Mancone, C., Tripodi, M., Maione, R., and Amati, P. (2009). Mitochondrial localization of PARP-1 requires interaction with mitofilin and is involved in the maintenance of mitochondrial DNA integrity. J. Biol. Chem. *284*, 31616–31624.

Ryu, K.W., Nandu, T., Kim, J., Challa, S., DeBerardinis, R.J., and Kraus, W.L. (2018). Metabolic regulation of transcription through compartmentalized NAD^+ biosynthesis. Science *360*, eaan5780.

Sallin, O., Reymond, L., Gondrand, C., Raith, F., Koch, B., and Johnsson, K. (2018). Semisynthetic biosensors for mapping cellular concentrations of nicotinamide adenine dinucleotides. eLife *7*, 7.

Schraufstatter, I.U., Hyslop, P.A., Hinshaw, D.B., Spragg, R.G., Sklar, L.A., and Cochrane, C.G. (1986). Hydrogen peroxide-induced injury of cells and its prevention by inhibitors of poly(ADP-ribose) polymerase. Proc. Natl. Acad. Sci. USA *83*, 4908–4912.

Selivanov, V.A., Zeak, J.A., Roca, J., Cascante, M., Trucco, M., and Votyakova, T.V. (2008). The role of external and matrix pH in mitochondrial reactive oxygen species generation. J. Biol. Chem. *283*, 29292–29300.

Sena, L.A., and Chandel, N.S. (2012). Physiological roles of mitochondrial reactive oxygen species. Mol. Cell *48*, 158–167.

Smeitink, J.A., Zeviani, M., Turnbull, D.M., and Jacobs, H.T. (2006). Mitochondrial medicine: a metabolic perspective on the pathology of oxidative phosphorylation disorders. Cell Metab. *3*, 9–13.

Sreelatha, A., Yee, S.S., Lopez, V.A., Park, B.C., Kinch, L.N., Pilch, S., Servage, K.A., Zhang, J., Jiou, J., Karasiewicz-Urbańska, M., et al. (2018). Protein AMPylation by an Evolutionarily Conserved Pseudokinase. Cell *175*, 809–821.e19.

Stein, L.R., and Imai, S. (2012). The dynamic regulation of NAD metabolism in mitochondria. Trends Endocrinol. Metab. *23*, 420–428.

Strømland, Ø., Niere, M., Nikiforov, A.A., VanLinden, M.R., Heiland, I., and Ziegler, M. (2019). Keeping the balance in NAD metabolism. Biochem. Soc. Trans. *47*, 119–130.

Symersky, J., Osowski, D., Walters, D.E., and Mueller, D.M. (2012). Oligomycin frames a common drug-binding site in the ATP synthase. Proc. Natl. Acad. Sci. USA *109*, 13961–13965.

Szczesny, B., Brunyanszki, A., Olah, G., Mitra, S., and Szabo, C. (2014). Opposing roles of mitochondrial and nuclear PARP1 in the regulation of mitochondrial and nuclear DNA integrity: implications for the regulation of mitochondrial function. Nucleic Acids Res. *42*, 13161–13173.

Toledo, L.I., Altmeyer, M., Rask, M.B., Lukas, C., Larsen, D.H., Povlsen, L.K., Bekker-Jensen, S., Mailand, N., Bartek, J., and Lukas, J. (2013). ATR prohibits replication catastrophe by preventing global exhaustion of RPA. Cell *155*, 1088–1103.

Vizcaíno, J.A., Csordas, A., del-Toro, N., Dianes, J.A., Griss, J., Lavidas, I., Mayer, G., Perez-Riverol, Y., Reisinger, F., Ternent, T., et al. (2016). 2016 update of the PRIDE database and its related tools. Nucleic Acids Res. *44* (D1), D447–D456.

Voorneveld, J., Rack, J.G.M., Ahel, I., Overkleeft, H.S., van der Marel, G.A., and Filippov, D.V. (2018). Synthetic α- and β-Ser-ADP-ribosylated Peptides Reveal α-Ser-ADPr as the Native Epimer. Org. Lett. *20*, 4140–4143.

Wacker, D.A., Ruhl, D.D., Balagamwala, E.H., Hope, K.M., Zhang, T., and Kraus, W.L. (2007). The DNA binding and catalytic domains of poly(ADP-ribose) polymerase 1 cooperate in the regulation of chromatin structure and transcription. Mol. Cell. Biol. *27*, 7475–7485.

Williamson, D.H., Lund, P., and Krebs, H.A. (1967). The redox state of free nicotinamide-adenine dinucleotide in the cytoplasm and mitochondria of rat liver. Biochem. J. *103*, 514–527.

Xu, Y., Zhou, P., Cheng, S., Lu, Q., Nowak, K., Hopp, A.K., Li, L., Shi, X., Zhou, Z., Gao, W., et al. (2019). A Bacterial Effector Reveals the V-ATPase-ATG16L1 Axis that Initiates Xenophagy. Cell *178*, 552–566.e520.

Zhang, Q., Piston, D.W., and Goodman, R.H. (2002). Regulation of corepressor function by nuclear NADH. Science *295*, 1895–1897.

CellPress
OPEN ACCESS

STAR★METHODS

KEY RESOURCES TABLE

REAGENT or RESOURCE	SOURCE	IDENTIFIER
Antibodies		
Anti-ADPr antibody	Hottiger Laboratory	N/A
Anti-pan-ADPr antibody	CST	Cat# E6F6ABF
Anti-PAR antibody	Enzo	Cat# ALX-210-890A-0100
Anti-ADPR eAF1521-FC	Hottiger Laboratory	Nowak et al., 2020
Anti-ATP5a antibody	Abcam	Cat# ab14748
Anti-γH2AX antibody	Biolegend	Cat# 613402
Chemicals, Peptides and Recombinant Proteins		
NAD^+	Sigma Aldrich	Cat# N7004-1g
ARTD8cat protein, recombinant	Hottiger Laboratory	N/A
ARTD10cat protein, recombinant	Hottiger Laboratory	N/A
ARTD1 protein, recombinant	Hottiger Laboratory	N/A
Dulbecco's modified Eagle's medium (DMEM)	ThermoFisher	Cat# 61965-026
Penicillin-streptomycin	ThermoFisher	Cat# 15140-122
Fetal calf serum	ThermoFisher	N/A
Blasticidin	InvivoGen	Cat# ant-bl-1
Hygromycin B	ThermoFisher	Cat# 10687010
Doxycycline hydrate	Sigma	Cat# D9891-1G
OptiMEM	ThermoFisher	Cat# 31985-047
Rotenone	Sigma Aldrich	Cat# R8875-5G
Myxothiazol	Sigma Aldrich	Cat# T5580
Antimycin	Sigma Aldrich	Cat# A8674-25mg
Oligomycin	Calbiochem	Cat# 495455
FCCP	Sigma Aldrich	Cat# C2910-10MG
PJ34	Adoog	Cat# A12665-50
Olaparib	Selleckchem	Cat# S1060
Talazoparib	Selleckchem	Cat# S7048
Niraparib	Adooq	Cat# A11026
Methyl Methanesulfonate	Sigma Aldrich	Cat# 129925-25G
Hydrogen peroxide solution	Sigma Aldrich	Cat# H1009-100ml
DAPI	BioLegend	Cat# 422801
Bovine serum albumin	Sigma Aldrich	Cat# A9418
Formaldehyde solution	Sigma Aldrich	Cat# F8775
Mowiol 4.88	Calbiochem	Cat# 475904
SiR-Halo	Lukinavičius et al., 2013	N/A
CP-TMR-C6-SMX	Sallin et al., 2018	N/A
Critical Commercial Assays		
Lipofectamine RNAiMAX	ThermoFisher	Cat# 13778-150
MultiScribe Reverse Transcriptase	ThermoFisher	Cat# 4311235
KAPA Biosystems Sybr Fast qPCR Kit	Roche	Cat# KK4600
Flp-In^{TM} TRex^{TM} Core Kit	ThermoFisher	Cat# K650001
Experimental Models: Cell Lines		
U-2 OS, human (female origin)	ATCC	Cat# HTB-96
C2C12, myoblasts, mouse	ATCC	Cat# CRL-1772

(*Continued on next page*)

Continued		
REAGENT or RESOURCE	SOURCE	IDENTIFIER
HeLa cells (female origin)	ATCC	Cat# CCL-2
U2OS 3xHA-EGFP-OMP25	This paper	N/A
U2OS Flp-In nuclear NAD^+ sensor; inducible	This paper	N/A
U2OS Flp-In cytoplasmic NAD^+ sensor; inducible	This paper	N/A
U2OS Flp-In mitochondrial NAD^+ sensor; inducible	This paper	N/A
Experimental Models: Organisms/strains		
C57BL/6N	Wellcome Trust Sanger Institute	N/A
Recombinant DNA		
pMXs-3xHA-EGFP-OMP25	Addgene	Cat# 83356
pCMV-VSV-G	Addgene	Cat# 8454
pUMVC	Addgene	Cat# 8449

RESOURCE AVAILABILITY

Lead Contact

Further information and requests for resources and reagents should be directed to and will be fulfilled by the Lead Contact, Michael O. Hottiger (michael.hottiger@dmmd.uzh.ch).

Materials availability

Proprietary material is available upon request from the authors.

Data and code availability

Image raw data and quantification data is available at Mendely Data:https://doi.org/10.17632/wp53bs344k.1. The mass spectrometry proteomics data have been deposited to the ProteomeXchange Consortium via the PRIDE (Vizcaíno et al., 2016) partner repository with the dataset identifier PXD013918. Other raw data is available directly from the authors.

EXPERIMENTAL MODEL AND SUBJECT DETAILS

Cell lines

All cell lines used for this study were originally purchased from ATCC, grown under standard sterile cell culture conditions (humidified atmosphere, 5% CO_2) and routinely tested for mycoplasma.

Human U-2OS, HeLa, A549 and HEK293T cells were cultured in high glucose containing Dulbecco's modified Eagle's medium (DMEM) supplemented with 5% penicillin/ streptomycin (P/S) and 10% (v/v) fetal calf serum (FCS).

Human U-2 OS 3xHA-EGFP-OMP25 cells were grown in high glucose containing DMEM supplemented with 5% P/S and 10% (v/v) FCS in the presence of 15 μg/mL Blasticidin to maintain selection pressure.

Stable U2OS Flp-In T-Rex cells expressing inducible NAD^+ sensor constructs were cultured in high glucose containing DMEM supplemented with 5% P/S and 10% (v/v) FCS in the presence of 100 μg/mL hygromycin and 15 μg/mL Blasticidin and 200 ng/mL doxycycline was added over-night if sensor expression was required.

Murine C2C12 cells were grown in high glucose and pyruvate containing DMEM, supplemented with 5% P/S and 20% (v/v) FCS and maintained below confluency.

METHOD DETAILS

Cell culture

Cell transfection and viral transduction

Lentiviruses were produced by co-transfecting HEK293T cells with viral constructs, packaging- and envelop plasmid in a ratio of 1:1 (construct: packaging/envelop) and 8:1 (packaging: envelop). using calcium phosphate. Roughly 12 h after transfection, the medium was removed and replaced with fresh DMEM. 2 days after transfection, the medium was collected and filtered through a 0.45 μm

mesh, to eliminate residual cells. Roughly 40% confluent U2OS cells were transduced with the virus-containing medium together with 5 mg/mL polybrene. After 24 h of recovery, cells were subjected to Blasticidin selection.

The Flp-In T-Rex System (ThermoFisher Scientific) was used to generate inducible U2OS Flp-In cell lines that express the NAD^+ sensors in the cytoplasm, nucleus or mitochondria, respectively. Cells were co-transfected with the pcDNA5/FRT/TO plasmid containing the gene of interest and the Flp recombinase (pOG44).

Drug treatments

Unless otherwise stated, cells were treated with the following compounds at the indicated concentrations and for the indicated amount of time: NMN (10 mM) for 1 h; Rotenone (3 or 10 μM), Myxothiazol (5 μM), Antimycin (10 μM), Oligomycin (5 μM) for 1 h; FCCP (2 μM) for 15 min; H_2O_2 (1 mM) for 10 - 60 min; MMS (1:10000) for 3 h; PJ-34 (10 μM), Olaparib (10 μM), Talazoparib (10 nM) and Niraparib (10 μM) for 30 min; PARG inhibitor (PDD00017273, 10 μM) for max 3 h, CHX (50μM) for up to 3 h. For co-treatments, cells were pre-treated with Rotenone, Myxothiazol, Antimycin or Oligomycin for 1 h or FCCP for 15min, prior to being treated with H_2O_2 for another 10 min. In other co-treatment experiments, 30 min after treatment with either PJ-34, Olaparib or Veliparib, FCCP was added for another 15 min, before the medium was removed and replaced with PBS only or PBS containing H_2O_2 for 10 min.

siRNA transfection

siRNA mediated knockdown of NAMPT, NMNAT-1, −2 and −3,SLC12A8 or SelO was performed via reverse transfection using Lipofectamine RNAi MAX according to the manufacturer's manual. In brief, the day after seeding cells into a 6 cm dish, at roughly 40% confluence, 25 nM siRNA were mixed with 5 μl lipofectamine in 500 μl serum-free OptiMEM and incubated for 20 min at room temperature (RT) before being added drop-wise unto the cells. 2 to 3 days after siRNA transfection, downstream experiments were performed. A scrambled siRNA was used as control for each experiment.

Immunostaining

For all IF experiments, cells were grown on 12 mm glass coverslips, treated if required, fixed in 4% FA for 15 min at RT and permeabilized for 10 min at RT in PBS supplemented with 0.2% Triton X-100 (Sigma Aldrich). If nuclear pre-extraction was required, cells were incubated in ice-cold permeabilization solution for 2 min before being fixed. Cells were blocked in filtered PBS supplemented with 0.1% Triton X-100 and 2% BSA for 1 h. The primary antibodies were further diluted in the same buffer and cells were incubated with the antibody solution over night at 4°C. In case of the anti-PAR antibody (Enzo), DMEM supplemented with 10% FCS was used for blocking and dilution of the primary antibody, which was incubated on the cells for 2 h at RT. All secondary antibodies were diluted in the standard blocking solution (0.1% Trition-X100 and 2% BSA in PBS) for 1 h at RT. After each antibody incubation, cells were washed 3 times with PBS. Following the last wash, cells were incubated with 0.1 μg/mL DAPI in PBS for 20 min at RT. After an additional PBS wash, the coverslips were briefly washed in distilled water and mounted on glass slides using 5.5 μl Mowiol solution per coverslip. The following antibodies were used for IF at the indicated concentrations: mouse anti-ATP5a (Abcam, 1:250), rabbit anti-PAR (Enzo, 1:1000), rabbit anti-pan ADPr (CST, 1:5000), mouse anti-γH2AX (biolegend, 1:500), rabbit anti-PARP1 (CST, 1:1000), mouse fusion FC-eAF1521 (1:400, our lab), rabbit anti-ADPr antibody (This paper, 1:500).

Confocal microscopy

Confocal images were acquired on an automated CLSM – Leica SP8 upright confocal laser scanning microscope, equipped with 4 solid state diode lasers (405, 488, 552 and 638 nm), using an HCX PL APO CS2 63x immersion oil objective. For all images, brightness and contrast were adjusted using FIJI. For all images within one experiment, the same acquisition and image processing settings were used.

ELISA

To test the binding of the newly generated anti-ADPr antibody toward ADP-ribosylation, ADP-ribosylated peptides (Abplanalp et al., 2017) were diluted in coating buffer (50 mM Na-Carbonate, pH 9.6), 100 μl of each diluted peptide was added to 1 well of a flat bottom microtiter plate each, and incubated over night at 4°C. After being washed 3 times with TBS-T, 200 μl blocking solution (5% milk in TBS-T) was added to each well and incubated for 1.5 h at room temperature. Thereafter, the blocking buffer was exchanged with 100 μl dilution buffer (0.5% milk in TBS-T) and 150 μl of the antibody to be tested (e.g., anti-ADPr antibody; 1:500) was added to one well. After 2 h of incubation at RT, the plate was washed 3x with TBS-T and subsequently, 100 μl of the diluted secondary antibody (IgG HRP; 1:1000) was added to each well and incubated for 50 min at RT before the wells were washed another 3 times in TBS-T. For signal detection, 50 μl TMB substrate solution were added to each well and, after 5 min, 100 μl of 1 M H_2SO_4 were added to stop the reaction. Finally, the signal intensity was evaluated using a Tecan plate reader at 540 nm.

In vitro ADP-ribosylation assay

For auto-modification of ARTD8- and ARTD10 cat, as well as ARTD1 10 pmol of the respective recombinant protein were incubated in reaction buffer (50 mM Tris-HCl pH 7.4, 4 mM $MgCl_2$ and 250 μM dithiothreitol (DTT)) with 10 μM NAD^+ for 30 min at 37°C.

For the detection of isolated PAR chains, ARTD1 was auto-modified as described above, and PAR isolated using a proteinase K digest for 2 h at 42°C. Subsequently, remaining PAR chains were purified by loading the digest on a NucleoSpin Gel and PCR clean-up column.

Rapid isolation of mitochondria from cells

Rapid isolation of mitochondria from U2OS 3xHA-EGFP-OMP25 cells was performed as previously described (Chen et al., 2016). In brief, roughly 15*10^6 cells were trypsinized, washed once in PBS and resuspended in 0.5 mL mitochondrial isolation buffer (MIB; 75 mM sucrose, 225 mM mannitol, 20 mM HEPES, 0.5 mM EDTA, pH 7.4), supplemented with 1x proteinase inhibitor cocktail (Roche) and 10 μM PJ-34. Cells were further homogenized with around 80 strokes of a 1 mL homogenizer with a Teflon head, and the homogenate was spun down at 1000 g for 2 min in order to discard nuclei and unbroken cells. Further, the supernatant was incubated with 100 μl pre-washed HA-beads for 3.5 min in an end-over-end rotor and subsequently washed twice with MIB. For detergent lysis, the beads were incubated with 150 μl of lysis buffer (50 mM Tris-HCl, pH 7.4, 150 mM NaCl, 1 mM EDTA, 1% Triton X-100), supplemented with the same inhibitors used for the MIB. Protein quantification of the lysates was performed using the BCA assay according to the manufacturer's protocol.

Western- and Dot blotting

For WB analysis, proteins were separated via SDS-page on a 12% SDS-polyacrylamide gel at 120V. A wet-transfer into a PVDF membrane was performed at 30 V over-night and membranes were blocked with 5% milk in TBS-T for 1 h at RT. Primary antibodies were diluted in 1% milk in TBST and incubated at 4°C over-night. After 3 washes, the secondary antibody, diluted in TBST, was incubated for 1 h at RT. After another 3 washes, specific proteins/bands were visualized with the Odyssey infrared imaging system (LI-COR). The following primary and secondary antibodies were used for WB analysis at the indicated concentrations: rabbit anti-HA (Abcam, 1:1000), rabbit anti-GST Z5 (Santa-Cruz, 1:1000), rabbit anti-CoxIV (Abcam, 1:1000), mouse anti-ATP5a (Abcam, 1:1000), rabbit anti-ADPR (N/A, 1:500), rabbit anti-ADPR (CST, 1:5000), IRDye 800CW goat anti-rabbit IgG (1:15,000, LI-COR, P/N 925-32211), and IRDye 680RD Goat anti-Mouse IgG (1:15,000, LI-COR, P/N 925-68070). For dot blot analysis, auto-modified proteins or isolated PAR chains were vacuum-blotted onto a nitrocellulose membrane, that was further blocked in milk and stained with antibodies as described above.

ADPr-Peptide Enrichment

ADPr-Peptide enrichments were carried out as previously described (Leutert et al., 2018) with the following protocol modifications. Following PARG-mediated PAR-to-MAR peptide ADPr-modification reduction, the peptides were enriched using an Af1521 macrodomain affinity enrichment (Martello et al., 2016) for 2 h at 4°C. Following this enrichment, the peptide mixtures were enriched a second time as described above using an ADP-ribose affinity evolved Af1521 macrodomain (Nowak et al., manuscript submitted). Both enriched samples were then prepared for MS analysis as described previously (Martello et al., 2016).

QUANTIFICATION AND STATISTICAL ANALYSIS

Quantitative image-based cytometry (QIBC)

Automated multichannel wild-field microscopy for quantitative image-based cytometry was performed with the Olympus ScanR screening system equipped with an inverted motorized Olympus IX83 microscope, a motorized stage, IR-laser hardware autofocus, a fast emission filter wheel with single band emission filters, and a digital monochrome Hamamatsu ORCA-FLASH 4.0 V2 sCMOS camera (2048 × 2048 pixel, pixel size 6.5 μm x 6.5 μm, 12 bit dynamics) as previously described (Michelena et al., 2018). For each condition, a minimum of 1500 cells was acquired using the UPLSAPO 20x objective (NA 0.9). Images were taken under non-saturating conditions and identical settings were applied to all coverslips within the same experiment. Following acquisition, images were analyzed using the Olympus ScanR Image Analysis Software version 3.0.1. After a dynamic background correction was applied, image segmentation was performed based on the DAPI signal in order to identify cell nuclei as individual objects. Further, mitochondria were identified as associated objects using similar intensity-based segmentation based on ATP5a co-staining, within an area spaced minimally 1.6 μm and maximally 26 μm from the nuclear periphery. Mean fluorescence intensities within the nuclear or mitochondrial masks were quantified per cell and are displayed as cell population violin plots using GraphPad Prism 8.0. Untreated samples were arbitrarily set to 30 for visualization and normalization purposes and to allow statistical analyses across independent replicate experiments. Each QIBC-based ADP-ribosylation measurement was performed at least 3 times, and a representative graph from one experiment with ATP5a co-staining is shown. In addition, representative pictures, in which the individual channels have been adjusted for brightness and contrast to the same settings, were chosen to accompany the quantifications.

Statistical Analysis

For statistical analysis, the normalized mean fluorescence intensity of 3 to 5 independent experiments were compared using a Student's t test with *, $p < 0.05$; **, $p < 0.005$; ***, $p < 0.0005$.

Liquid Chromatography and Mass Spectrometry Analysis

Identification of ADP-ribosylated peptides from U2OS and mouse skeletal muscle was performed on an Orbitrap Fusion Tribrid mass spectrometer (Thermo Fisher Scientific), coupled to a nano EasyLC 1000 liquid chromatograph (Thermo Fisher Scientific) (Leutert et al., 2018). We applied an ADP-ribose product-dependent method called HCD-PP-EThcD (Bilan et al., 2017). Briefly, the method includes high-energy data-dependent HCD, followed by high-quality HCD and EThcD MS/MS when two or more ADP-ribose fragment peaks (136.0623, 250.0940, 348.07091, and 428.0372) were observed in the HCD scan. A detailed description of the MS parameters can be found in (Bilan et al., 2017).

Raw Mass Spectrometry Data Analysis

RAW MS files were converted to mzXML using ReAdW (https://github.com/PedrioliLab/ReAdW). Each mzXML file was split into 2 files, that exclusively contained HCD35 or ETD MS/MS scans, using an in-house script. At the same time, ADP-ribosylation diagnostic ions (i.e., 136.0623, 250.094, 348.07091, 428.0372) were also removed from the MS/MS scans. These files were then searched against the Swiss-Prot mouse protein database version of 2018-09 or Swiss-Prot Human protein database version of 2019-01, depending on the origin of the sample. Comet (http://comet-ms.sourceforge.net/) version 2018.01 rev. 2 was used for these searches and the following variable modifications were accounted for: 15.9949 at M; 541.061110 at S, D, E, R, K, Y, H, C, T; 42.010565 at the N terminus; and 57.021464 at C. Semi-tryptic peptides with a maximum of 4 missed cleavages and a precursor tolerance of 25 ppm were considered. Peptide (PeptideProphet) and protein (ProteinProphet) probabilities were assigned using TPP v5.0.0 Typhoon (https://sourceforge.net/projects/sashimi/). Finally, to derive the list of ADP-ribosylated mitochondrial proteins and peptides, the search results were filtered as follows: i) the protein list was filtered at 1% FDR; ii) the peptides associated with the remaining proteins were filtered at 1% FDR; iii) non-mitochondrial proteins were removed; and iv) non-ADP-ribosylated peptides were removed.

Intracellular, compartmentalized NAD^+ measurements

Sensor expression in stable U2OS Flp-In cells was induced overnight via administration of 200 ng/mL doxycycline. The day following induction, cells were labeled with 500 nM CP-TMR-C6-SMX and 500 nM SiR-Halo again overnight. After labeling, cells were washed at least three times with DMEM to thoroughly remove any excess of the dyes and subjected to the desired treatment (e.g., H_2O_2 or Rotenone). Finally, cells were trypsinzed and the FRET ratio, as a proxy for NAD^+ levels, was analyzed via flow cytometry using the LRS II Fortessa. The following laser and filter combination was used: 405 nm to acquire foreword- and sideward scatter, 561 (586/15) to acquire the donor signal (CP-TMR-C6-SMX), 561 (635 LP, 670/30) to acquire the FRET signal and 640 (670/14) to acquire the acceptor (SiR-Halo) only. The data were analyzed using FlowJo. The FRET ratio was calculated by dividing the FRET signal by the donor signal.

Molecular Cell, Volume *81*

Supplemental Information

Mitochondrial NAD^+ Controls

Nuclear ARTD1-Induced ADP-Ribosylation

Ann-Katrin Hopp, Federico Teloni, Lavinia Bisceglie, Corentin Gondrand, Fabio Raith, Kathrin Nowak, Lukas Muskalla, Anna Howald, Patrick G.A. Pedrioli, Kai Johnsson, Matthias Altmeyer, Deena M. Leslie Pedrioli, and Michael O. Hottiger

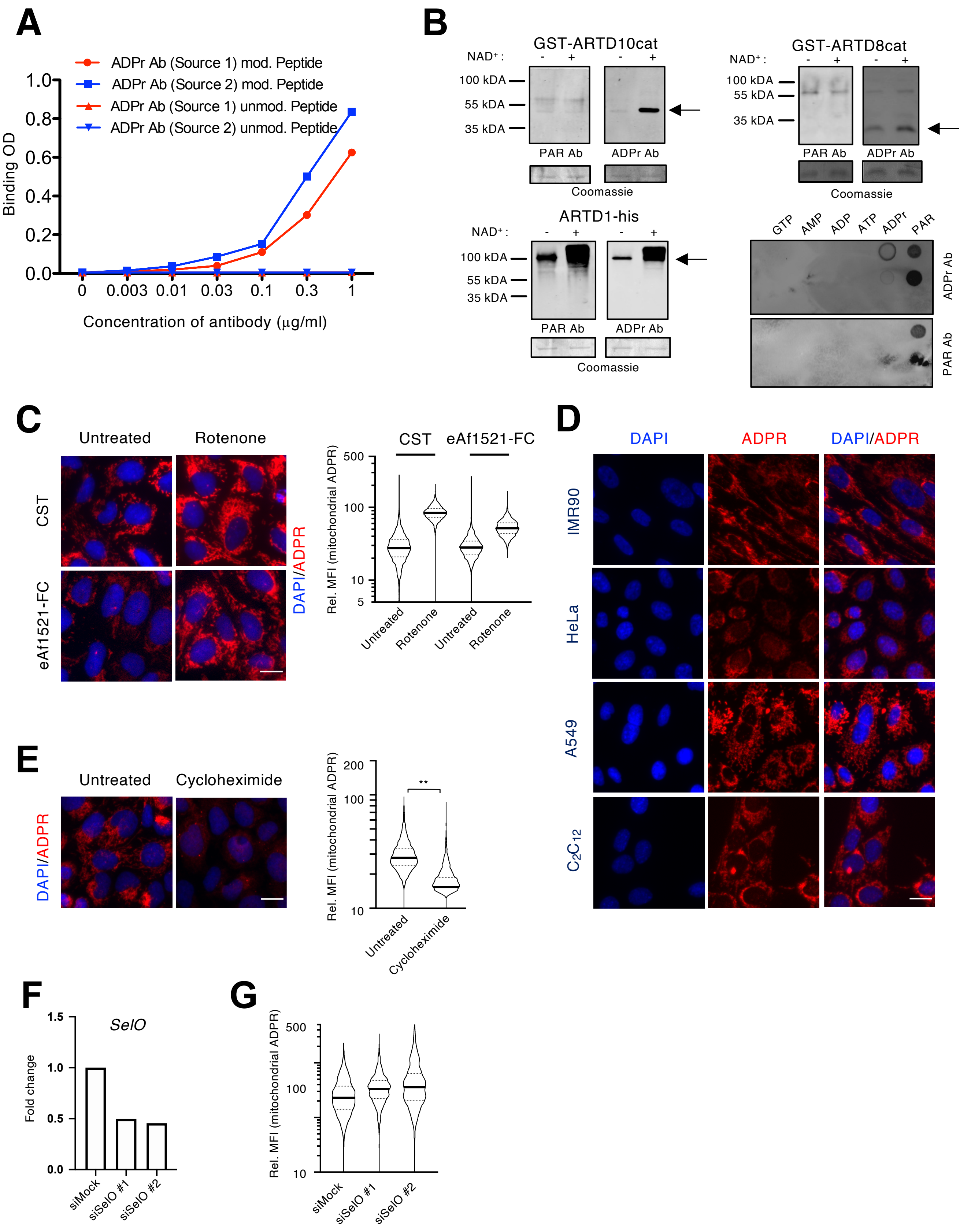

A
ADPr Ab (Source 1) mod. Peptide
ADPr Ab (Source 2) mod. Peptide
ADPr Ab (Source 1) unmod. Peptide
ADPr Ab (Source 2) unmod. Peptide
Binding OD
Concentration of antibody (µg/ml)
B
GST-ARTD10cat
GST-ARTD8cat
ARTD1-his
NAD⁺:
PAR Ab
ADPr Ab
Coomassie
GTP AMP ADP ATP ADPr PAR
C
Untreated
Rotenone
CST
eAf1521-FC
DAPI/ADPR
Rel. MFI (mitochondrial ADPR)
D
DAPI
ADPR
DAPI/ADPR
IMR90
HeLa
A549
C2C12
E
Untreated
Cycloheximide
F
SelO
Fold change
siMock
siSelO #1
siSelO #2
G

Suppl. Figure 1: Validation of the new pan-anti-ADP-ribosylation antibody, related to Figure 1

(**A**) ELISA using the two anti ADPr antibodies (source 1 and source 2) in titrating concentrations on two different modified and unmodified peptides. (**B**) WB of the auto-modified catalytic domain of ARTD10 (ARTD10-cat), or ARTD8 (ARTD8-cat) or auto-modified ARTD1 stained with the anti-PAR antibody and the anti-ADPr antibody. Dot blot with two concentrations (10 and 1 μM; upper and lower row, respectively) of GTP, AMP, ADP, ATP, ADPr and PAR, stained with the anti-ADPr antibody or an anti-PAR antibody (lower right panel). (**C**) IF staining of U2OS cells either untreated or treated with 3 μM Rotenone using either a commercial anti-pan-ADPr antibody or an engineered Af1521-FC fusion protein. (**D**) IF staining using the anti-ADPr antibody (red) on C2C12 myoblasts, HeLa, A549 and IMR90. (**E**) IF staining for ADP-ribosylation in cells pre-treated with cycloheximide. (**F**) qPCR analysis of the knockdown levels of SelO. (**G**) Quantification of mitochondrial ADP-ribosylation in U2OS after knockdown of SelO with two independent siRNAs. For the representation of all quantifications the signal for every event was normalized over the mean of the control/untreated sample, which was arbitrarily set to 30. The Y-axes of all violin plots are depicted as log 10 scales. Scale bars indicate 20 μm. For statistical analysis, a Student's t test was performed ($n = 3-5$; $^{*}p < 0.05$; $^{**}p < 0.005$; $^{***}p < 0.0005$).

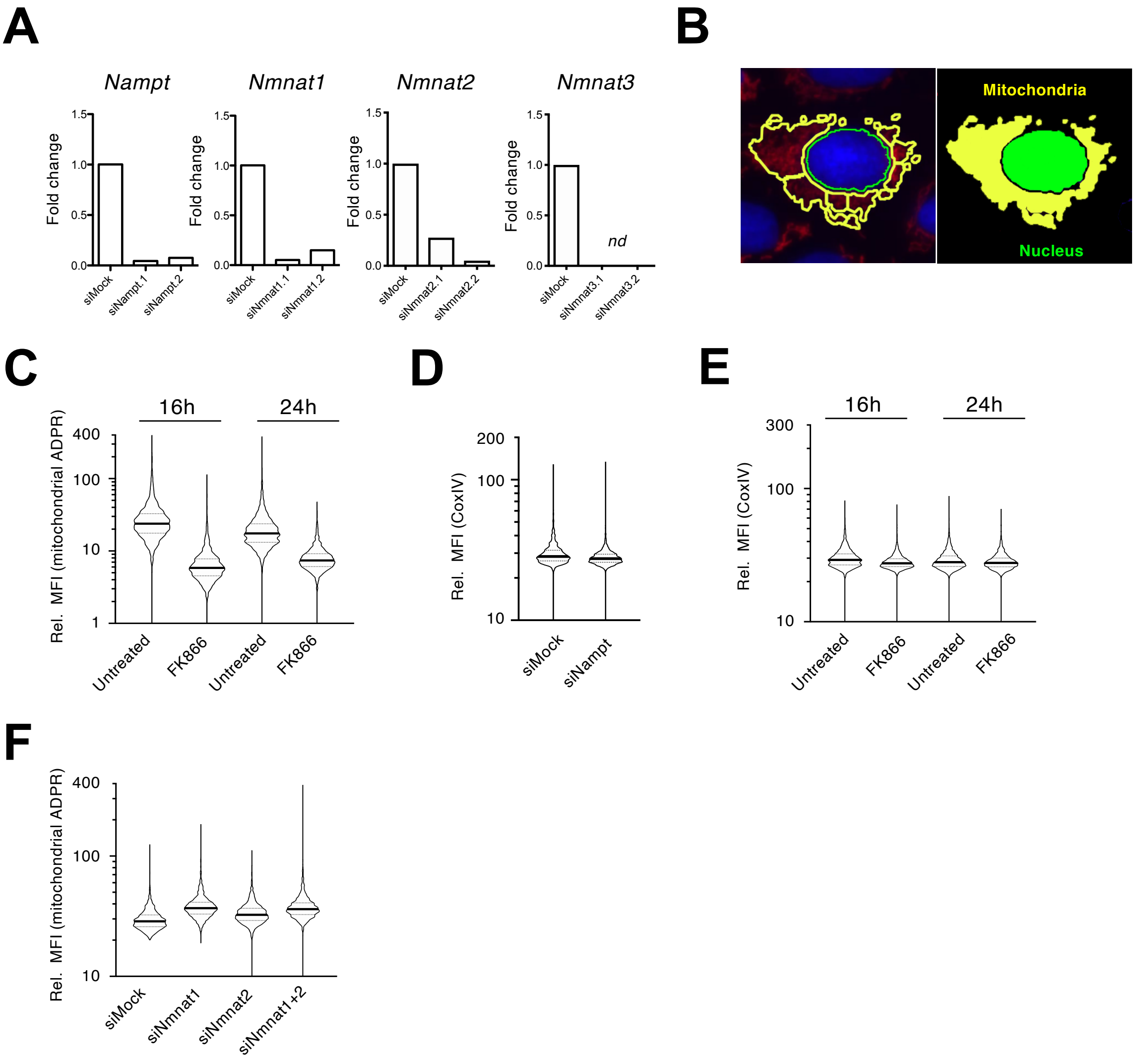

Suppl. Figure 2: Characterization of mitochondrial ADP-ribosylation, related to Figure 1

(A) qPCR analysis of the knockdown levels of NAMPT, NMNAT1, NMNAT2 and NMNAT3 after siRNA mediated knockdown of the respective genes in U2OS cells. **(B)** Scheme of the masks applied to quantify nuclear- as well as extra-nuclear IF signals. **(C)** Quantification of mitochondrial ADP-ribosylation after treatment with the NAMPT inhibitor FK866 for 16 or 24h. **(D,E)** Quantification of the mitochondrial COXIV signal in U2OS cells after knockdown of NAMPT or treatment with FK866. **(F)** Quantification of mitochondrial ADP-ribosylation in U2OS cells after knockdown of NMNAT1, NMNAT2 or both. For the representation of all quantifications the signal for every event was normalized over the mean of the control/untreated sample, which was arbitrarily set to 30. The Y-axes of all violin plots are depicted as log 10 scales.

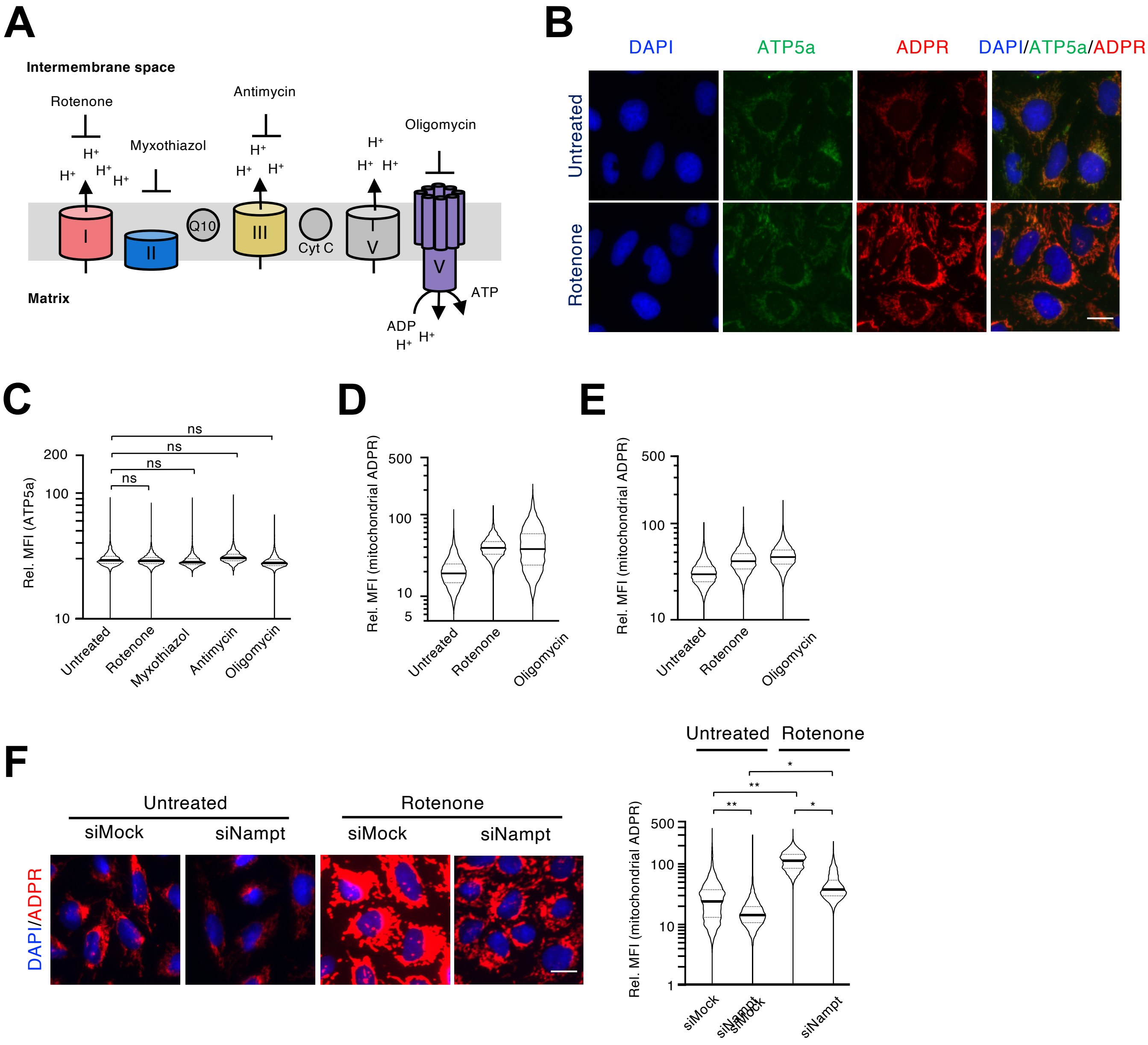

Suppl. Figure 3: Inhibition of respiration increases mitochondrial ADP-ribosylation, related to Figure 1

(A) Schematic overview of the respiratory chain, including the commonly used inhibitors Rotenone, Myxothiazol, Antimycin and Oligomycin. **(B)** Co-IF staining of ADP-ribosylation (anti-ADPr antibody; red) together with the ATP5a antibody (green) in untreated or Rotenone treated U2OS cells. **(C)** Quantification of the mitochondrial COXIV signal in U2OS cells after treatment with different respiratory chain inhibitors. **(D,E)** Quantification of mitochondrial ADP-ribosylation in A549 (**D**) and HeLa (**E**) cells after treatment with Rotenone or Oligomycin. **(F)** IF for ADP-ribosylation (anti-ADPr antibody; red) of U2OS subjected to 3 days of knockdown of NAMPT and subsequent Rotenone treatment. Representative pictures for each condition are shown on the left while the mean fluorescence intensity (MFI) of the mitochondrial ADP-ribosylation is shown on the right. The quantifications of all signals (mitochondrial and nuclear) were normalized as described in Fig.1. For the representation of all quantifications the signal for every event was normalized over the mean of the control/untreated sample, which was arbitrarily set to 30. The Y-axes of all violin plots are depicted as log 10 scales. Scale bars indicate 20 µm. For statistical analysis, a Student's t test was performed (n = 3-5; *p < 0.05; **p< 0.005; ***p < 0.0005).

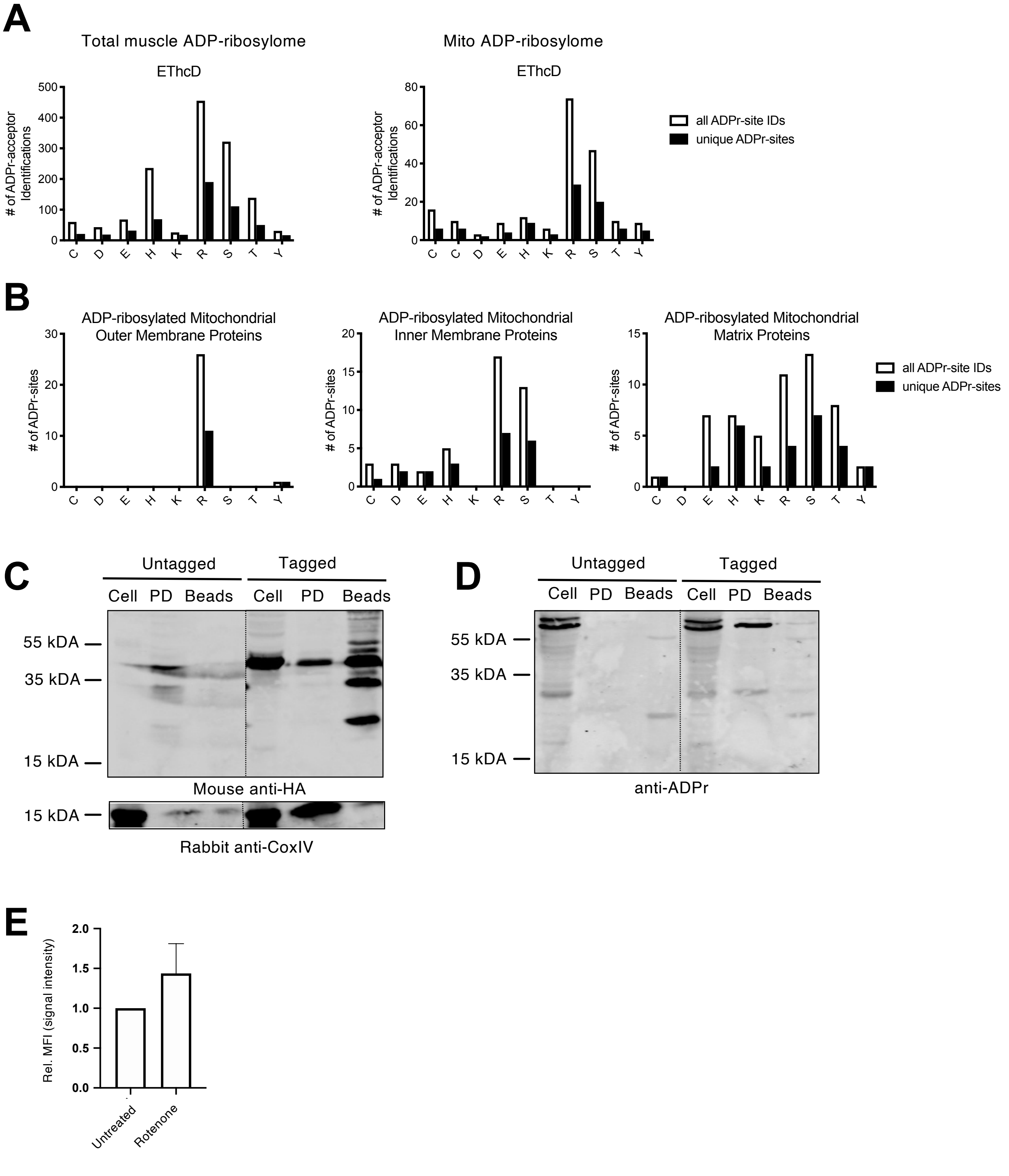

Suppl. Figure 4: Various mitochondrial inner- and outer membrane proteins are ADP-ribosylated, related to Figure 2

(A) Potential EThcD-defined amino acid acceptor site distributions identified in the total muscle ADP-ribosylome and the mitochondria-specific ADP-ribosylome. **(B)** Potential EThcD-defined amino acid acceptor site distributions on ADP-ribosylated mitochondrial proteins that localize to the outer membrane (left panel), the inner membrane (middle panel) or the matrix (right panel). **(C)** Pull down control of Fig. 2D: WB on mitochondrial lysates from U2OS cells stably transfected with HA-tagged OMP25 using an anti-HA- and an anti-COXIV antibody. **(D)** WB of mitochondrial lysates from U2OS cells stably transfected with HA-tagged OMP25 using the anti-ADPr antibody. **(E)** Quantification of the ADP-ribosylation signal intensity of Fig. 2E.

Suppl. Figure 5

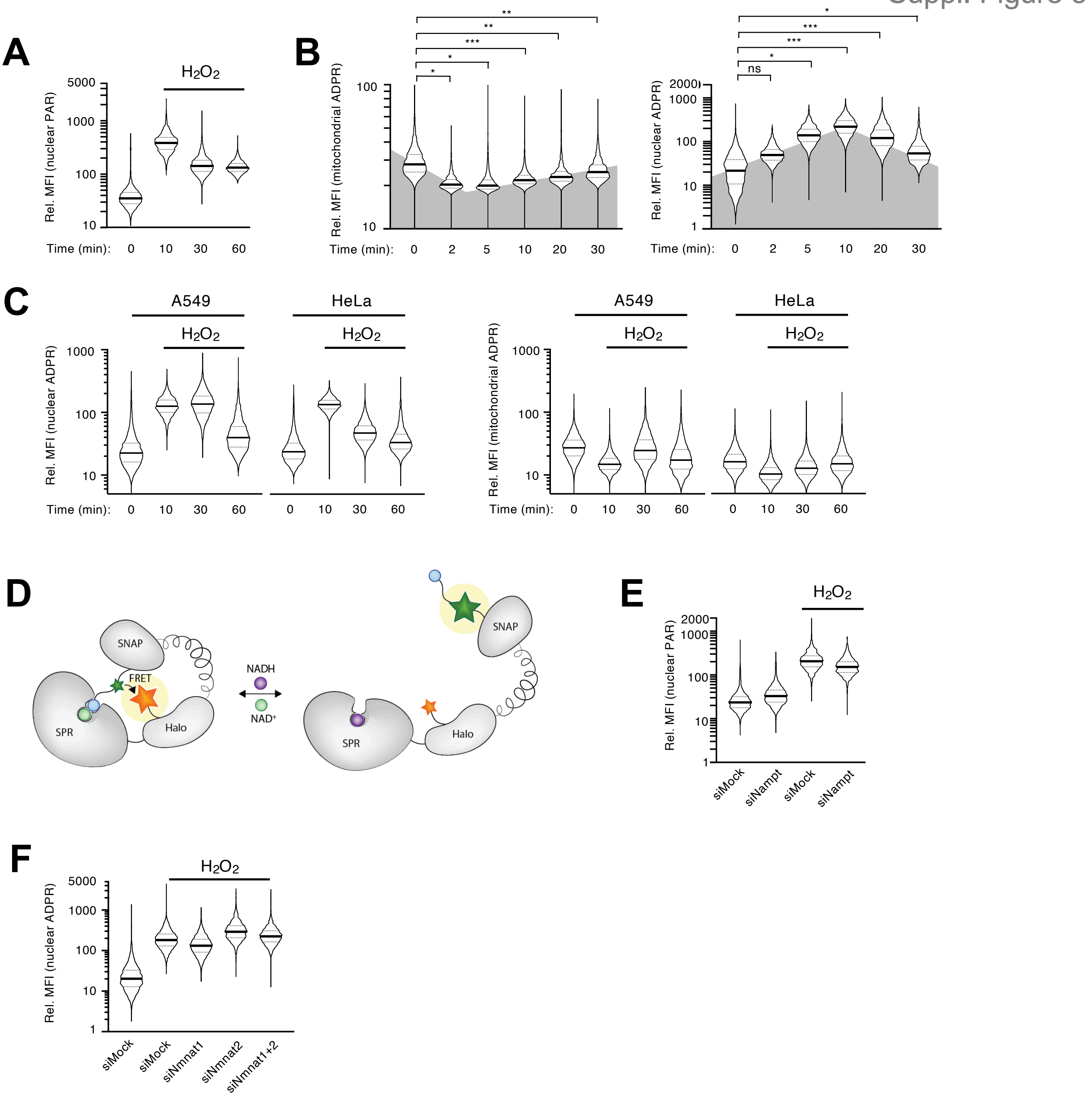

Suppl. Figure 5: Mitochondria-derived NAD$^+$ is crucial for the formation of nuclear PAR, related to Figure 3
(A,B) Quantification of the nuclear PAR **(A)** and nuclear as well as mitochondrial ADP-ribosylation **(B)** of the U2OS cells at various timepoints after H_2O_2 treatment. (**C**) Quantification of nuclear and mitochondrial ADP-ribosylation in A549 and HeLa cells at different time points after H_2O_2 treatment. **(D)** Visualization of the FRET-sensor constructs used for intracellular NAD$^+$ measurements. Scheme adapted from (Sallin et al., 2018). **(E)** U2OS cells were transfected with siRNA targeting NAMPT, treated with H_2O_2 and nuclear and nuclear ADP-ribosylation analyzed by IF using an anti-PAR antibody. **(F)** Quantification of nuclear ADP-ribosylation following 10 min of H_2O_2 treatment of U2OS after siRNA knockdown of NMNAT1, NMNAT2 or both together. The quantifications of all signals (mitochondrial and nuclear) were normalized as described in Fig. 1 and the Y-axes of all plots are depicted as log10 scale.

Suppl. Figure 6

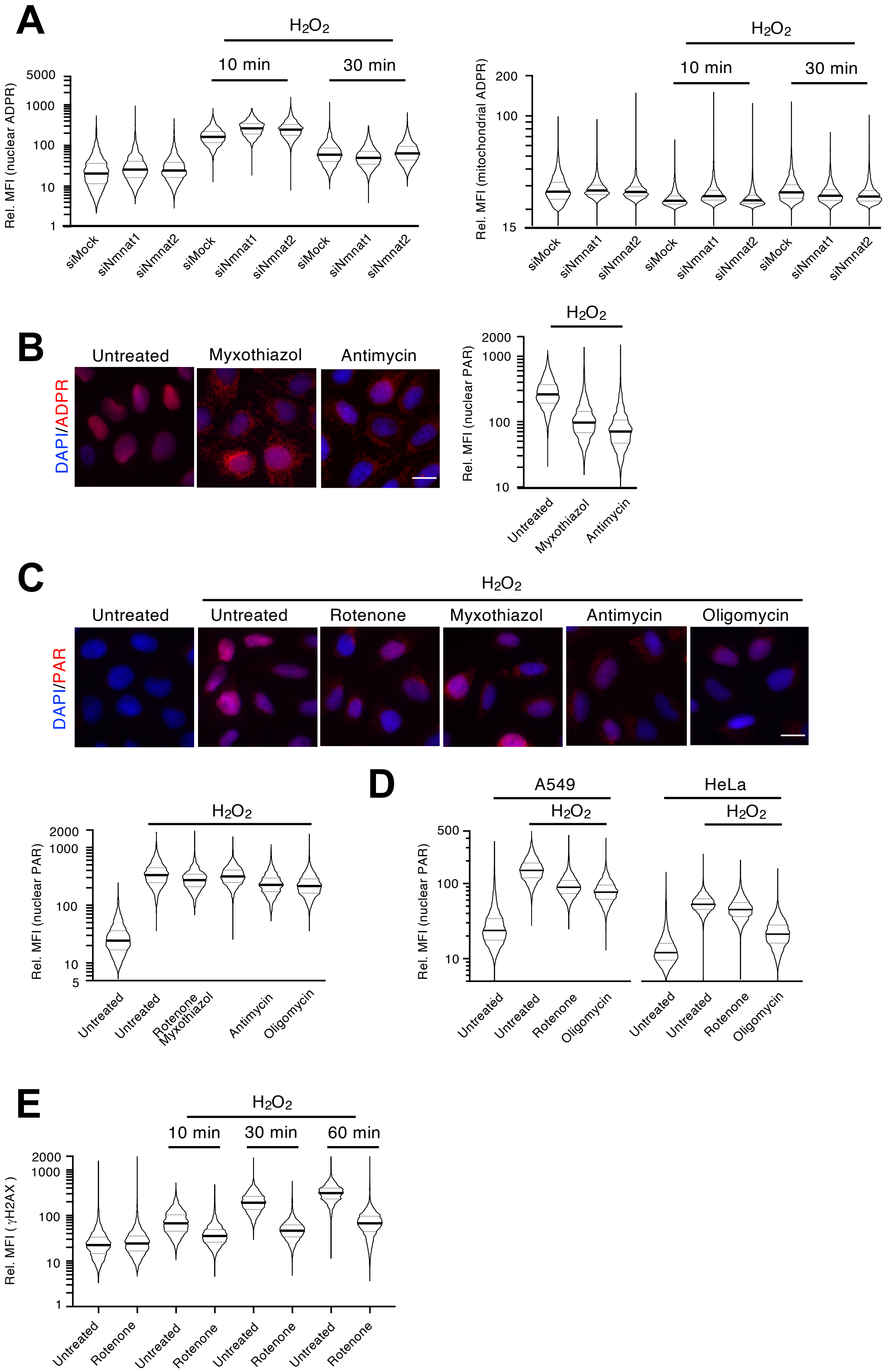

Suppl. Figure 6: Effect of NMNAT1/2 Knockdown on cellular ADP-ribosylation and Rotenone on H_2O_2 induced γH2AX formation, related to Figure 4

(A) U2OS cells were transfected with siRNA targeting NMNAT1 or 2 and nuclear (left panel) and mitochondrial (right panel) ADP-ribosylation was analyzed via IF and quantified at various timepoints following H_2O_2 treatment. **(B,C)** U2OS cells were pre-treated with either Rotenone, Myxothiazol, Antimycin or Oligomycin prior to a treatment with H_2O_2 and subsequent analysis of nuclear ADP-ribosylation via IF using an anti-ADPr antibody (**B**) or an anti-PAR antibody (**C**) . (**D**) A549 and HeLa cells were pre-treated with either Rotenone or Oligomycin before being treated with H_2O_2 and analyzed via IF. **(E)** U2OS cells were treated with H_2O_2 and Rotenone for the indicated time points and γH2AX levels were assessed via IF and quantified. The quantifications of all signals (mitochondrial and nuclear) were normalized as described in Fig. 1 and the Y-axes of all plots are depicted as log10 scale. Scale bars indicate 20 μm.

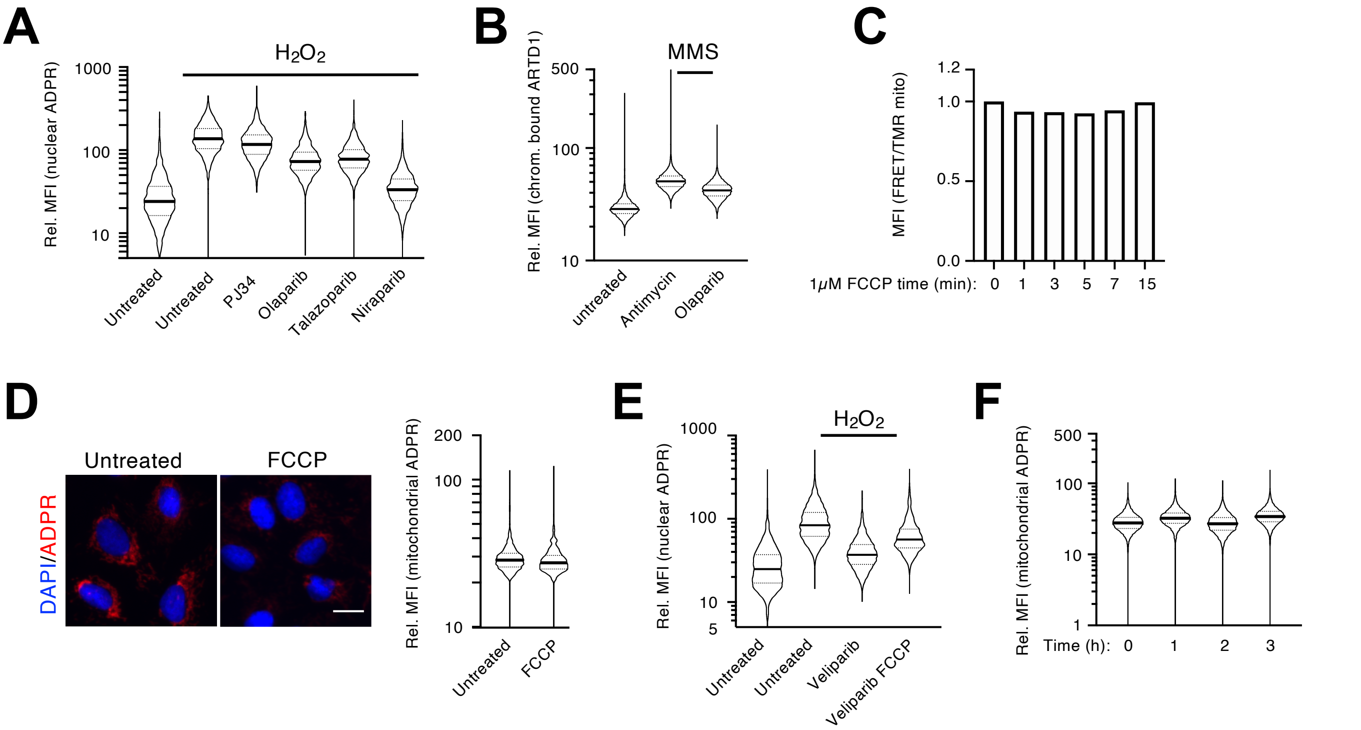

Suppl. Figure. 7: Effect of PARP inhibitors on nuclear and FCCP or PARG inhibitors on mitochondrial ADP-ribosylation, related to Figure 5

(A) U2OS cells were pre-treated for 1h with various PARP inhibitors and subsequently treated for 10 min with H_2O_2. **(B)** U2OS cells were co-treated with MMS and Antymicin for 2h and following pre-extraction, chromatin-bound ARTD1 was analyzed via IF. **(C)** Mitochondrial NAD^+ levels following FCCP treatment were assessed in stable U2OS Flp-In™ T-Rex™ cells expressing inducible NAD^+ sensors and analyzed via flow cytometry. Increased FRET ratios correspond to increased NAD^+ levels. **(D)** U2OS cells were treated with FCCP and mitochondrial ADP-ribosylation was analyzed via IF with the anti-ADPr antibody and quantified. (**E**) U2OS cells were pre-treated with Veliparib, then subjected to an FCCP pulse followed by H_2O_2 and analyzed via IF using an anti-ADPr antibody. (**F**) U2OS cell were treated with PARG inhibitor for the indicated time and mitochondrial ADP-ribosylation analyzed via IF using an anti-ADPr antibody. The quantifications of all signals (mitochondrial and nuclear) were normalized as described in Fig. 1 and the Y-axes of all plots are depicted as log10 scale. Scale bars indicate 20 μm.
